# Targeting the Translocator Protein (18 kDa) in Cardiac Diseases: State of the Art and Future Opportunities

**DOI:** 10.1021/acs.jmedchem.3c01716

**Published:** 2023-12-19

**Authors:** Emma Baglini, Valeria Poggetti, Chiara Cavallini, Debora Petroni, Francesca Forini, Giuseppina Nicolini, Elisabetta Barresi, Silvia Salerno, Barbara Costa, Patricia Iozzo, Danilo Neglia, Luca Menichetti, Sabrina Taliani, Federico Da Settimo

**Affiliations:** § Department of Pharmacy, University of Pisa, Via Bonanno 6, Pisa 56126, Italy; ¥ Institute of Clinical Physiology, National Research Council of Italy, CNR Research Area, Via G. Moruzzi 1, Pisa 56124, Italy; £ Fondazione CNR/Regione Toscana Gabriele Monasterio, Cardiovascular and Imaging Departments, CNR Research Area, Via G. Moruzzi 1, Pisa 56124, Italy

## Abstract

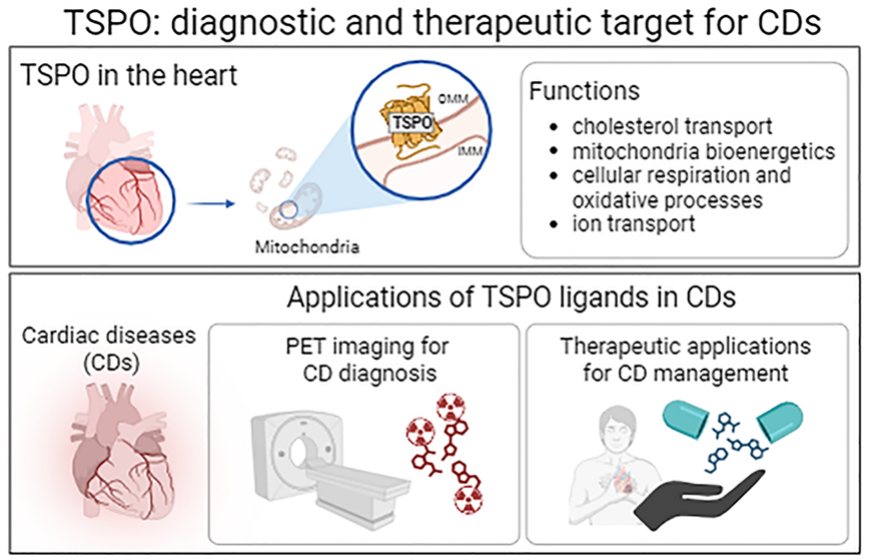

Mitochondria dysfunctions are typical hallmarks of cardiac disorders (CDs). The multiple tasks of this energy-producing organelle are well documented, but its pathophysiologic involvement in several manifestations of heart diseases, such as altered electromechanical coupling, excitability, and arrhythmias, is still under investigation. The human 18 kDa translocator protein (TSPO) is a protein located on the outer mitochondrial membrane whose expression is altered in different pathological conditions, including CDs, making it an attractive therapeutic and diagnostic target. Currently, only a few TSPO ligands are employed in CDs and cardiac imaging. In this Perspective, we report an overview of the emerging role of TSPO at the heart level, focusing on the recent literature concerning the development of TSPO ligands used for fighting and imaging heart-related disease conditions. Accordingly, targeting TSPO might represent a successful strategy to achieve novel therapeutic and diagnostic strategies to unravel the fundamental mechanisms and to provide solutions to still unanswered questions in CDs.

## 1 Introduction

Despite significant advances in the treatment of cardiovascular diseases (CVDs), the latter remain the leading causes of morbidity, disability, and death, with very high social and economic impact. CVDs are estimated to affect 471 million people worldwide with approximately 17.6 million deaths per year (32% of all global deaths), a trend that will increase to 24 million by 2030, which means 66 000 deaths per day.^1^ In Europe, according to data from the fifth edition of the European Cardiovascular Disease Statistics, CVDs affect more than 80 million people (48% men and 52% women) and are responsible for 3.9 million deaths per year (45% of all causes of death). Among CVDs, heart failure (HF) is the leading cause of hospitalization in people over 65 and has very high mortality rates: 1 in 25 patients does not survive the first admission, 10% die within 30 days, and 30% die within a year of admission. Accordingly, it is always a challenge and a relevant priority to discover new diagnostic and therapeutic targets for cardiac disorders (CDs). Among them, the translocator protein (18 kDa), TSPO, is an attractive emerging candidate. TSPO is an integral membrane protein of 169 amino acids that is composed of five transmembrane α-helical domains. In most tissues, it is predominantly expressed in mitochondria, more specifically at the contact site between the outer mitochondrial membrane (OMM) and the inner mitochondrial membrane (IMM).^2^ The participation of mitochondrial TSPO in a multimeric complex known as mitochondrial permeability transition pore (mPTP) along with other proteins, including the 32 kDa voltage-dependent anion channel (VDAC) and the 30 kDa adenine nucleotide translocase (ANT), is supported by some,^3^ but not all, studies.^4−7^ A nonmitochondrial localization of TSPO has also been described, *e.g.*, in the plasma membrane of erythrocytes, the nuclear and extranuclear fraction of breast cancer cells, the nuclei of human hepatocytes, and other organelle membranes of several cell types.^8^

TSPO is evolutionarily well-conserved, and it is present in almost all organisms, suggesting a critical role in biological processes.^9^ Regarding tissue distribution, TSPO is ubiquitous, but it is mainly found in steroid-synthesizing tissues (including adrenal glands and gonads), kidneys, nasal epithelium, lungs, and the heart, while a lower expression is shown in the brain and liver.^8^

TSPO participates in several cellular functions, but one of the most frequently described is the regulation of the rate-limiting step of steroidogenesis, explaining its prominent localization at the level of steroid-synthesizing tissues. Specifically, TSPO is involved in the internalization of cholesterol into the mitochondrion. In the IMM, cholesterol is converted into pregnenolone by the enzyme CYP11A1 (cytochrome P-450 family 11 subfamily A member 1), then pregnenolone is transported to the sarcoplasmic reticulum where it is metabolized to steroid products.^8^ It has been hypothesized that TSPO ligands stabilize the tertiary structure of the protein and consequently facilitate mitochondrial import of cholesterol, leading to increased steroidogenic efficiency in various steroidogenic *in vitro* models.^10^ Interestingly, it has been demonstrated that the equilibrium thermodynamic parameter predicting the steroidogenic efficacy of a TSPO ligand is the residence time to the protein rather than the binding affinity.^11^ TSPO is also involved in other processes related to mitochondrial bioenergetics, such as apoptosis,^12^ cellular respiration and oxidative processes,^13^ mitochondrial metabolism,^14^ protein import,^15^ ion transport,^16^ immunomodulation,^17^ porphyrin transport, and heme biosynthesis.^18^

TSPO is upregulated in several cancerous tissues, including lung, ovary, colon, prostate, and brain cancers,^19^ as well as in activated microglial cells in neuropsychiatric pathologies such as neurodegenerative diseases (*i.e.*, Alzheimer’s and Parkinson’s diseases). Due to this feature, TSPO has been suggested as a biomarker of neuroinflammation^20^ and the progression of these pathologies.^21^ Conversely, TSPO is downregulated in the brains of patients with post-traumatic stress, anxiety, and obsessive-compulsive disorders^22^ and upon repeated stress, including noise exposure.^23^ Small synthetic TSPO ligands have been developed and biologically evaluated as potential tools for treating TSPO-related disorders, and TSPO has gained recognition as a marker and therapeutic target of neuropsychiatric pathologies.

**Figure 1:**
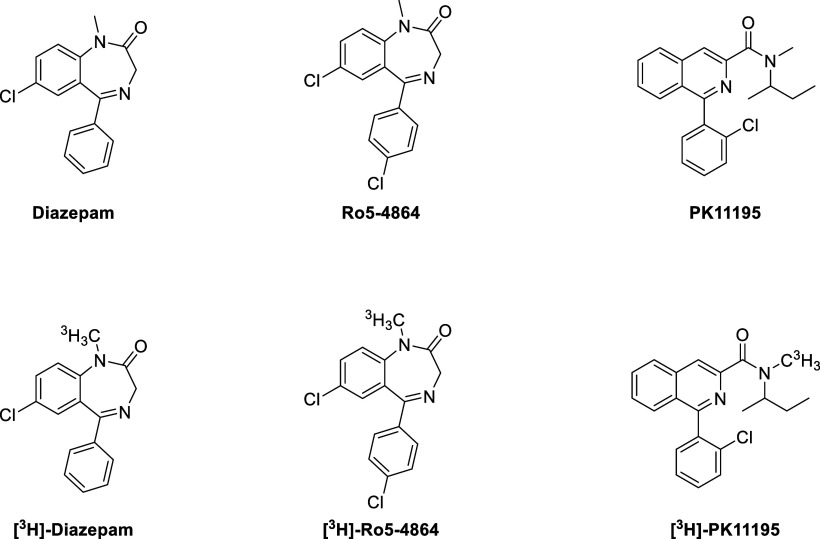
Chemical structures of diazepam, Ro5-4864, and PK11195 and their corresponding [^3^H]-radiolabels.

Biodistribution studies (*in vivo* and *in vitro*) using radiolabeled ligands, *e.g.*, [^3^H]-diazepam (rat and guinea-pig cardiac membranes),^24^ [^3^H]-Ro5-4864 (7-chloro-5-(4-chlorophenyl)-1-methyl-1,3-dihydro-2*H*-benzo[*e*][1,4]diazepin-2-one), and [^3^H]-PK11195 (*N*-methyl-*N*-(1-methylpropyl)-1-(2-chlorophenyl)isoquinoline-3-carboxamide) (Figure 1), demonstrated TSPO is also located in the heart, prevailing in the myocardial ventricles compared to atria.^25^

TSPO is up-regulated upon acute electroshock in cardiac ventricles, followed by an increase in TSPO density in the cerebral cortex.^26^ Recently, TSPO has been found to be involved in CDs and, more specifically, in ischemia-reperfusion injury (IRI), but its exact role remains to be established.^27^

In the present report, an overview of the recent literature evaluating the effects of TSPO ligands on cardiac pathophysiology is presented, together with the most representative examples of the few TSPO radioligands studied so far for diagnosing CDs, particularly atherosclerosis, myocarditis, large vessel vasculitis (LVV), and myocardial infarction (MI). The discussion, moving from diagnostic to therapeutic applications, aims to shed light on the potential of TSPO as a target to be exploited by the scientific community to advance cutting-edge research in the field of CDs management.

## 2 The Role of TSPO in Cardiac Pathophysiology

Several studies demonstrated that TSPO ligands may have different cardiac effects, indicating a possible involvement of TSPO in regulating heart physiology. Initially, TSPO has been proposed to influence cardiac contractility and heart rate by modulating calcium flux and ion transport, respectively.^27,28^ TSPO ligands decrease the calcium current and thus lower calcium release from the sarcoplasmic reticulum, which might explain their observed negative inotropic effects.^29^ The negative chronotropic effect of TSPO ligands has been ascribed to a decreased inward ion current during the fourth phase of the action potential in pacemaker cells, which leads to a hyperpolarization of the resting membrane potential.^30^ Further studies have questioned the involvement of TSPO ligands in the regulation of cardiac electrophysiology because certain TSPO ligands are responsible for biological effects only at concentrations that exceed their affinity values for the target, and in these conditions they can also bind other molecular targets/receptors.^28^ Thus, their resulting pharmacological effect might hardly be attributable to their specific binding to TSPO. For example, the negative chronotropic effect of Ro5-4864 (Figure 1) might be a result of the direct interaction with calcium channels.^31^ It is conceivable that inconsistencies may depend on different experimental models, species, types of TSPO ligands, and concentrations used.^27^ It is important to remark that this complicated issue concerning synthetic TSPO ligands has been frequently ascribed to the complexity of this protein modulating numerous processes. It is not uncommon that a TSPO ligand can determine different effects with reference to a specific TSPO function depending on the experimental system employed. Thus, the classical concept of inhibitor (antagonist) and activator (agonist), commonly recognized for membrane ligand/receptor systems, cannot be applied to TSPO ligands and synthetic ligands, initially labeled as TSPO agonists or antagonists, often revealed to produce comparable effects if different experimental models are applied.

In addition, TSPO ligands have been proposed to exert a protective effect in CDs by reducing reactive oxygen species (ROS) production and preventing mitochondrial dysfunction and stress-dependent cardiomyocyte loss.^28^ Together with these possible effects, the main physiologic mechanisms involving TSPO that have been investigated are related to the interference with cholesterol transport and the interaction with other mitochondrial components, as detailed below.

### 2.1 TSPO, Cholesterol Transport, And Oxidative Stress

The first recognized and best-studied function of TSPO is to facilitate cholesterol transport from the cytosol to the IMM, a crucial step for steroidogenesis and other biological processes.^28^ However, in a pro-oxidative condition, cholesterol within mitochondria is highly sensitive to ROS attack and autoxidation, resulting in the formation of oxysterols, which may promote mitochondria damage, such as membrane lipid peroxidation, increased membrane permeability and, ultimately, cell death.^32−36^ The most abundant oxysterols in cardiac mitochondria are 7α- and 7β-hydroxycholesterol (OHC), 7-ketochol, and cholesterol epoxides, derived from 7-hydroperoxycholesterol. Among them, 7α-OHC, 7β-OHC, and 7-ketochol do not promote further lipid peroxidation, while hydroperoxycholesterol and cholesterol epoxides change the biophysical organization of lipids and proteins within membranes, thus affecting membrane fluidity.

During myocardial reperfusion following an ischemic event, cholesterol accumulates in the mitochondrion and generates self-oxidized oxysterols,^35,36^ which may be involved in the myocardial IRI. Accordingly, Musman et al. demonstrated that in the diabetic hypercholesterolemic rat model (ZDF fa/fa), the increased accumulation of cholesterol, other sterols, and oxysterols inside cardiac mitochondria dramatically exacerbated mitochondrial impairment following ischemia reperfusion (IR).^36^ A previous study showed hypercholesterolemia increases mitochondrial oxidative stress in the porcine myocardium.^37^ Thus, the correlation between hypercholesterolemia and mitochondrial damage in these experimental models underlines the possible deleterious effects of cholesterol-derived oxysterols.^35,36^ Besides propagating lipid peroxidation, oxysterols contribute to oxidative stress by depleting the antioxidant defense of cardiomyocytes, including glutathione. For example, 7-ketochol and 7β-OHC cause apoptosis via the mitochondrial pathway by reducing cell antioxidant activity. In addition, oxysterol can enhance the activity of superoxide-producing enzymes, such as NADPH oxidase and xanthine oxidase, further exacerbating oxidative stress.^34,38^

Interestingly, the inhibition of oxysterol accumulation as a consequence of cholesterol accumulation in the myocardial mitochondrial matrix through TSPO ligands was shown to exert cardioprotective effects and rescue oxidative phosphorylation in a lean and hypercholesterolemic murine model of IR, even if the underlying mechanism still needs to be established.^35,36^ However, the available findings suggest that oxysterols, which are produced in pathological conditions, could represent a link between mitochondrial damage, cholesterol accumulation, and the potential therapeutic role of TSPO ligands in CDs.

### 2.2 TSPO-Interacting Mitochondrial Components

TSPO has been shown to influence the activity of other mitochondrial components, such as the mPTP, VDAC, and inner membrane anion channel (IMAC), that play a crucial role in determining the fate of cardiomyocytes in acute and chronic CDs, such as acute MI and HF.^39,40^

#### 2.2.1 mPTP

The mPTP supramolecular complex is a nonspecific channel with a cutoff of 1.5 kDa that is formed and opens under stress conditions, triggering the so-called mitochondrial permeability transition that is responsible for cardiomyocyte death.^41^ The molecular identity of mPTP-forming units has long been debated, yet no definitive model has been agreed upon.^4−7^ Although the peptidyl-prolyl *cis–trans* isomerase (PPIase), or cyclophilin D (CypD), is the only component unambiguously involved in mPTP regulation, TSPO, among others, has been attributed at least an indirect facilitating role in channel opening. Cardiac IR is the main pathological condition able to trigger irreversible mPTP opening. Following ischemia, the lack of oxygen supply to the mitochondrial respiration chain blocks mitochondrial ATP synthesis. In the first few minutes of ischemia, anaerobic glycolysis copes with the lack of oxygen, but the ATP produced is insufficient to support cardiac activity. This process has several consequences, such as ionic imbalance and membrane depolarization. A cytosolic calcium overload occurs at the expense of a decrease in mitochondrial calcium concentration.^42^ At first, the decreased mitochondrial Ca^2+^ amount is helpful as it causes acidosis, which protects the heart from ischemic damage^43^ and prevents mPTP opening. However, conditions in favor of the latter are established, such as the decrease in mitochondrial membrane potential and the increase in ADP and Pi concentrations. If ischemia persists, the increased cytosolic Ca^2+^ concentration activates Ca^2+^-dependent enzymes that cause membrane destruction and cell death. Rapid coronary reperfusion is the only therapeutic strategy used to date in hospitals to treat ischemic events. However, reperfusion paradoxically causes so-called reperfusion injury. Indeed, the sudden supply of oxygen following a period of hypoxia promotes an excessive production of ROS, which is also increased by the calcium accumulated in the cytosol during ischemia. ROS are then transported into the mitochondrion once the membrane potential is restored. The increase in ROS and augmented calcium concentrations lead to an increase in mitochondrial membrane permeability, resulting in mPTP opening. As a consequence, the proton motive force is dissipated because of the inability of the IMM to act as a barrier toward protons; this causes the uncoupling of oxidative phosphorylation, which in turn results in ATP depletion and, therefore, in the exhaustion of cellular energy.^44^ The increased permeabilization of the IMM because of mPTP opening causes mitochondrial swelling,^45^ which is in turn augmented by several processes accompanying postischemic heart reperfusion, such as adenine nucleotide depletion, high phosphate concentration, and oxidative stress.^44^ The main driving force of mitochondrial swelling is the equilibration of all low-molecular-weight osmolytes between the cytosol and mitochondria, while proteins are retained within their respective compartments.^45^ Thus, the highest protein concentration in the matrix exerts an osmotic pressure resulting in swelling of the matrix compartment.^45^ Mitochondrial swelling, followed by OMM breaking, in turn leads to the release of pro-apoptotic signaling molecules and irreversible mitochondrial damage.^41^ However, if insufficient ATP levels are maintained, apoptotic death predominates over necrotic cell death.^45^

The possible role of mPTP in CDs was first studied by Crompton et al.,^46^ who showed that preventing the opening of the pore could represent a potential target for cardioprotection against myocardial IRI.^47^ Accordingly, many drugs used in cardioprotection trials, such as sangliferin A (SfA),^48^ cyclosporin A (CsA),^49,50^ 6-MeAla-CsA, 4-methyl-val-CsA, *N*-methyl-4-isoleucine-CsA (NIM811), and d-3-MeAla-4-EtVal-CsA (Debio-025), prevent mPTP opening during reperfusion.^51,52^ Further works showed that the beneficial effect induced by CsA is mediated by the inhibition of CypD,^53^ the 18 kDa matrix protein encoded by the nuclear PPIF gene.^49^ In detail, CypD has been proposed to sensitize mPTP by interfering with the function of ATP synthase, a putative component of the mPTP.^54^

Several studies, which will be discussed in more detail below, have reported that certain TSPO ligands act as cardioprotective agents by preserving the physiological function of mitochondria and preventing cell death.^55−57^ Other TSPO ligands have been shown to promote mPTP opening and apoptosis regardless of TSPO,^58−60^ thus suggesting that this protein does not directly intervene in pore regulation.^7^ Accordingly, it has emerged that TSPO ligands play an indirect role in mPTP opening by acting on oxidative stress and the production of ROS as pivotal triggers of pore opening.

#### 2.2.2 VDAC

The VDAC, also known as a mitochondrial porin, is found in the OMM, and one of its primary roles is the regulation of both the input and output of mitochondrial metabolites, ions, and nucleotides controlling the exchanges between mitochondria and the rest of the cell. The anion channel is closely associated with TSPO at the contact sites between the IMM and OMM.^61^ VDAC isoforms 1 and 3 actively participate in intrinsic cell death by forming a large flexible pore that allows the release of pro-apoptotic proteins such as cytochrome c (Cyt-c), apoptosis-inducing factor (AIF), Smac/DIABLO, and endonuclease G.^62^ Furthermore, VDAC is considered a target for proteins of the Bcl-2 family^62^ and promotes apoptosis by favoring ROS overproduction in conjunction with TSPO activity.^63,64^ VDAC is also involved in mitochondrial damage and rupture since its closure causes a defect in ATP/ADP exchange, leading to mitochondrial swelling and breakage followed by the release of several proapoptotic factors such as Cyt-c into the cytosol, as observed during mPTP opening.^62^

TSPO ligands may exert their cardioprotective action by increasing the stabilization of the antiapoptotic Bcl-2 in the mitochondrial membrane at the expense of the proapoptotic Bax, thus restraining cell death.^56^ In particular, TSPO ligands hinder the interaction of pro-apoptotic proteins with VDAC at contact sites between IMM and OMM, where TSPO, VDAC, ANT and other proteins are located,^65^ thus preventing Cyt-c release, but also limiting the production of ROS and therefore the permeabilization of the OMM.

#### 2.2.3 IMAC

The IMAC is a partially anion-selective channel of the IMM found in both the heart and liver. Although its molecular identity is still unknown, its presence was first characterized in 1986 by Garlid and Beavis, who demonstrated that anions like Cl^–^, Br^–^, SO_4_^2–^, PO_4_^3–^, *etc*., could cross the mitochondrial membrane.^66^ The IMAC is mainly involved in mitochondrial volume homeostasis and also plays a role in the contractile and electrical functions of the heart. It has been implicated in postischemic damage by promoting the generation of arrhythmias during the reperfusion period.^67,68^ This occurs due to ROS production, particularly due to a mechanism known as “ROS-induced ROS release” (RIRR). Briefly, RIRR is a process in which an initial release of ROS by a cellular compartment due to oxidative stress triggers the production and the release of ROS in other compartments.^69^ Regarding cardiac damage, IMAC is thought to be activated by the production of ROS at the mitochondrial level, which in turn causes the efflux of superoxide anions.^70,71^ In a study conducted in 2003,^72^ it was shown that RIRR can trigger oscillations of mitochondrial NADH and oscillatory depolarization of the IMM in cardiomyocytes, which in turn may cause arrhythmias in the reperfusion phase following ischemia by altering myocyte excitability.^67^ In addition, it has been shown that IMAC opening due to RIRR also influences mPTP opening.^73,74^ In particular, the pore opening is triggered by the oxidation of thiols in cardiomyocytes.^74^ IMAC does not participate in forming complexes with TSPO. However, TSPO ligands limit mitochondrial ROS production avoiding the threshold for IMAC opening to be reached, which represents an additional mechanism for indirect modulation of mPTP and limitation of arrhythmias.^67,68,72,75,76^ TSPO ligands have also been shown to inhibit IMAC activation in swelling assays on isolated mitochondria,^77^ strengthening the assumption that they are functionally related to each other, even though through a still incompletely understood mechanism. However, some controversy emerged, especially considering the concentrations of ligands necessary to inhibit IMAC and perform cardioprotective action (>30 μM), which far exceed far the affinity range in the nanomolar order.^66,67^

## 3 Cardiac Imaging via TSPO Radioligands

In clinical practice, molecular cardiac imaging serves a significant role in clinical cardiology.^78,79^ It enables a detailed evaluation of the pathophysiology of heart injury and the subsequent remodeling, which is essential in developing new and effective therapies.^80^ One of the well-established clinical applications of positron emission tomography (PET) in cardiac imaging is the assessment of myocardial perfusion with high accuracy in the absolute measurement of myocardial blood flow and coronary flow reserve. This application promises to be expanded thanks to the availability of novel compounds like [^18^F]-flurpiridaz.^81^ Another established clinical application of cardiac PET is the assessment of myocardial and vascular glucose metabolism with the use of the radiopharmaceutical leader in PET imaging, *i.e.*, [^18^F]-FDG (2-[^18^F]-fluoro-2-deoxy-d-glucose), even if the quality of the PET signal, in particular in the wall of vessels, is still limited. Nevertheless, PET has multiple potentials in cardiac and vascular molecular imaging.

TSPO is one of the most interesting inflammatory biomarkers exploited for PET imaging.^82^ Nonetheless, thus far there are just a few TSPO radioligands employed for cardiac imaging purposes, representing a new and valuable opportunity to exploit the potential of PET imaging, particularly thanks to the quantification capabilities.

**Chart 1:**
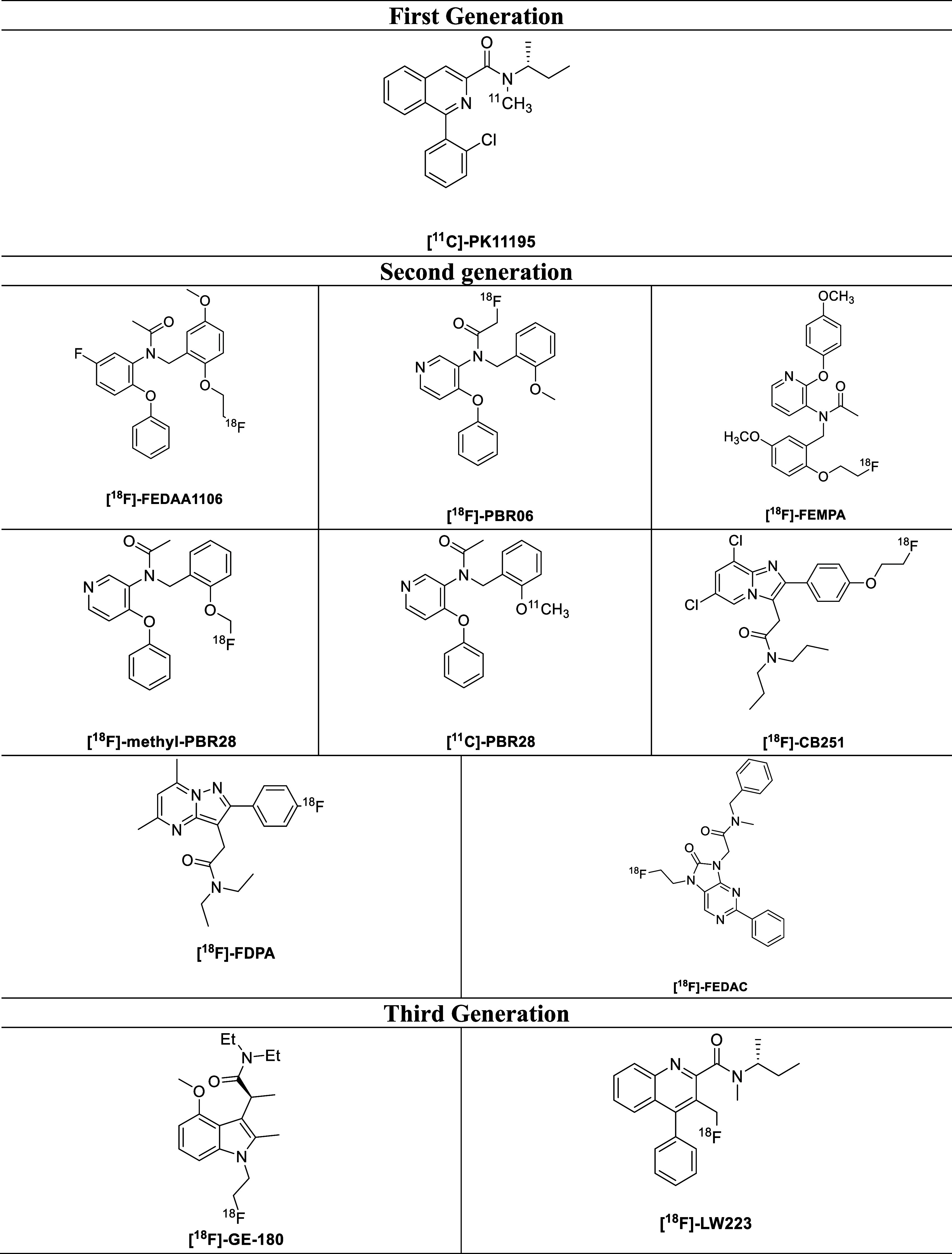
TSPO Radiotracers Tested in PET Cardiology Imaging

A list of TSPO PET radiotracers tested in cardiology imaging is reported in Chart 1. Most of these were initially developed for neuroimaging applications due to TSPO’s high expression in activated microglial cells characterizing neuroinflammatory diseases.^82,83^

### 3.1 First-Generation PET Tracers for TSPO

PK11195 and Ro5-4864 (Figure 1) belong to the so-called first-generation TSPO ligands.^84^ In 1984, PK11195 was radiolabeled with carbon-11^85^ to give [^11^C]-PK11195 (Chart 1, Table 1), the first radiotracer for TSPO used in PET imaging. This radiotracer was used for imaging atherosclerosis,^86^ a chronic disease of large and medium-sized arteries involving inflammatory processes that leads to major CVDs, such as ischemic heart disease, stroke, and peripheral vascular disease.^87^ Macrophages play a crucial role in atherosclerosis onset, since they are recruited in the vessel wall from the beginning of the inflammatory process and participate in plaque progression and/or rupture;^88^ in a nutshell, they are the primary inflammatory cell types in atherosclerotic plaques. Because activated macrophages express high TSPO levels,^89^ TSPO radioligands could represent valuable tools for detecting inflammation associated with atherosclerosis by PET. Experiments performed in an atherosclerotic mouse model showed [^11^C]-PK11195 uptake in inflamed plaques but similar accumulation in unaffected arterial walls, discouraging a potential application in the clinical field.^90^

**Table 1:**
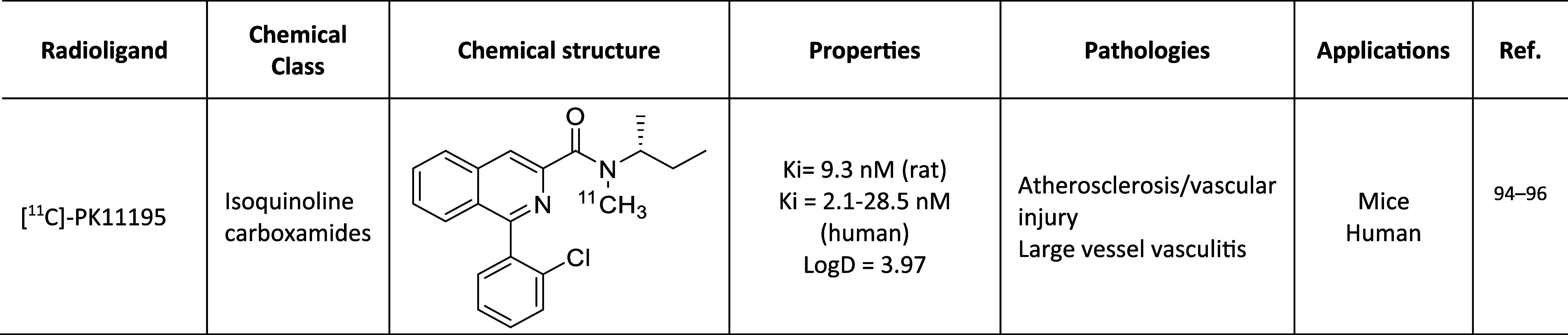
First-Generation PET Tracer for TSPO (95,96)

In addition, a clinical study performed in patients with abdominal aortic aneurysms showed that chronic inflammation of the vessel wall was not detectable with [^11^C]-PK11195.^91^

More promising results were obtained in a proof-of-concept clinical study regarding the imaging of intraplaque inflammation with the dual-modal PET/[^11^C]-PK11195/contrast-enhanced computed tomography (CT) angiography, where the possibility to distinguish recently active and symptomatic plaques from asymptomatic plaques was highlighted.^86^

The same dual modality was used for imaging vascular inflammation in patients with LVV (*e.g.*, giant cell arteritis or Takayasu’s arteritis),^92^ a chronic granulomatous inflammatory condition occurring in the aorta vessel wall or its main branches.^93^

The radiotracer allowed the assessment of arterial inflammatory activity in LVV patients, distinguishing patients with active and nonactive or more quiescent disease.^92^ PET/CT angiography could detect [^11^C]-PK11195 uptake in the vascular wall, providing anatomical details on vessel wall thickening and excluding atherosclerotic disease.^92^

Nonetheless, the promising results of these experiments were accompanied by some drawbacks. The high level of nonspecific binding,^94^ due to the high lipophilicity of the compound (Table 1), involves a weak signal-to-noise ratio that hampers its quantification, and the short half-life of carbon-11 (20 min) limits the clinical application of [^11^C]-PK11195. To overcome these obstacles, alternative TSPO probes were developed.

### 3.2 Second-Generation PET Tracers for TSPO

These new tracers were conceived to obtain compounds with lower lipophilicity than the previous generation, maintaining a high affinity for TSPO (Chart 1, Table 2). The labeling was performed mainly using fluorine-18, having a longer half-life (109.7 min) than carbon-11 and therefore being more easily manageable.

**Table 2:**
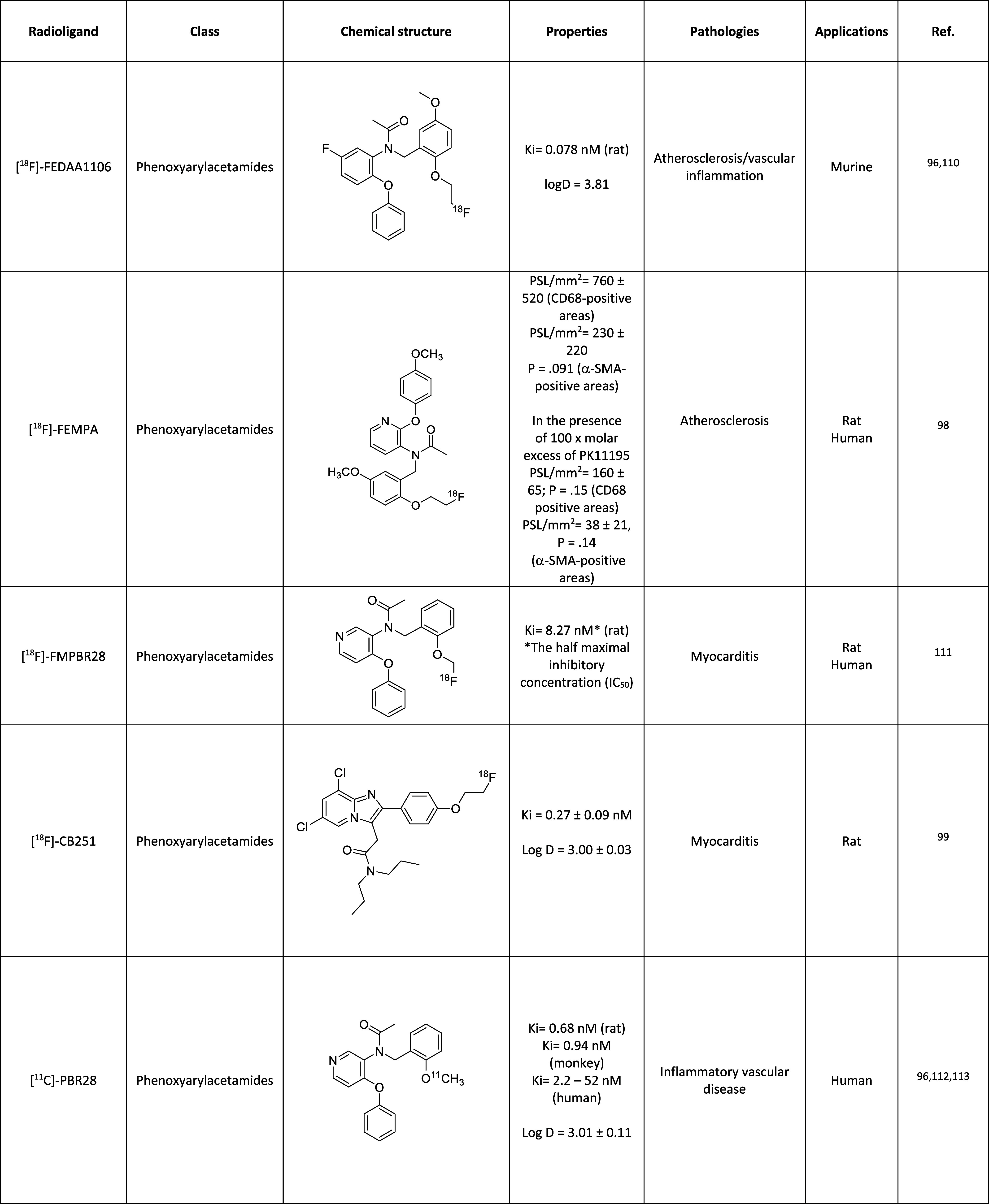

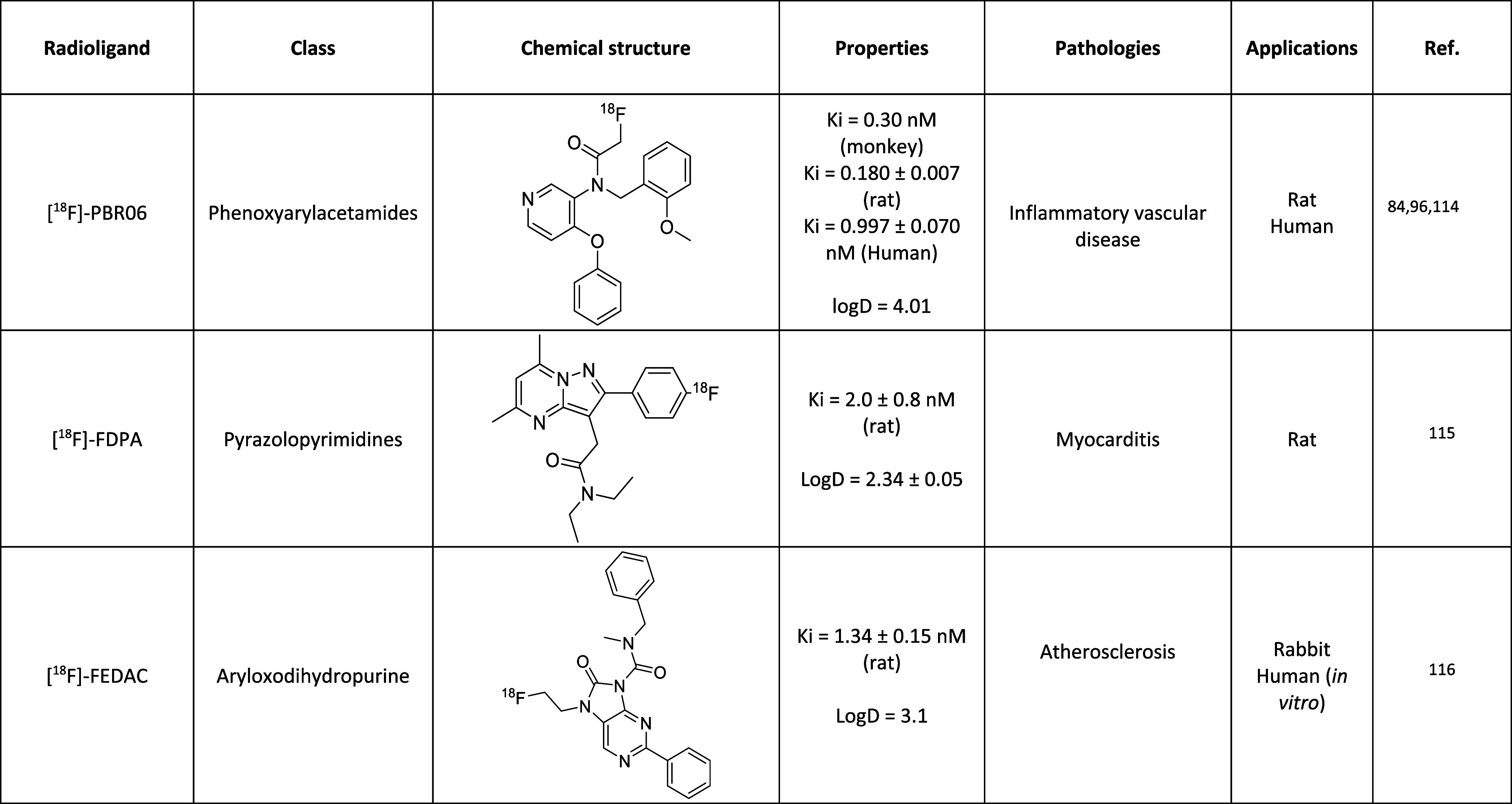
Second-Generation PET Tracers for TSPO (110,111,112,113,114,115,116)

One of these new compounds is [^18^F]-FEDAA1106 [*N*-(5-fluoro-2-phenoxyphenyl)-*N*-(2-^18^F-fluoroethyl-5-methoxybenzyl) acetamide] (Chart 1, Table 2), which was used to image vascular inflammation *in vivo* in a murine model.^97^ Furthermore, the same authors compared this TSPO radioligand with [^18^F]-FDG for PET imaging of vascular inflammation, showing that the [^18^F]-FEDAA1106 signal was significantly higher at the inflamed, disturbed flow region than the noninflamed, uniform flow regions. In contrast, differences in [^18^F]-FDG uptake were less distinct.

Significant tracer uptake in lesion areas was observed; however, the murine model has an induced and intense local inflammation in the vessel wall, which may not appear equally in a clinical situation of atherosclerotic patients. Thus, subsequent studies used alternative approaches to better capture variability in inflammatory activity in plaques, as seen in clinical atherosclerosis.

When one benzene ring of DAA1106 was replaced with a pyridine ring, a series of phenoxyarylacetamide derivatives with a lower lipophilicity was produced (Chart 1, Table 2).

In 2016, Hellberg et al. investigated [^18^F]-FEMPA (*N*-{2-[2-^18^F-fluoroethoxy]-5-methoxybenzyl}-*N*-[2-(4-methoxyphenoxy)pyridin-3-yl]acetamide) (Chart 1, Table 2) as a radiotracer for the detection of atherosclerotic plaque inflammation^98^ in the mouse aorta. Similar to [^11^C]-PK11195, the [^18^F]-FEMPA uptake ratio between the atherosclerotic plaque and the nonatherosclerotic vessel wall was not favorable; therefore, the authors proposed that this radiotracer may be more suitable for imaging intense areas of inflammation, such as LVV.

[^18^F]-Fluoromethyl-PBR28 (*N*-(2-((fluoro-^18^F)methoxy)benzyl)-*N*-(4-phenoxypyridin-3-yl)acetamide) and [^18^F]-CB251 (2-(2-(4-(2-[^18^F]fluoroethoxy)phenyl)-6,8-dichloroimidazo[1,2-*a*]pyridin-3-yl)-*N*,*N*-dipropylacetamide) (Chart 1, Table 2) were used in a comparative study to evaluate their suitability for myocarditis diagnosis.^99,100^ Myocarditis is an inflammatory myocardial condition that, in the acute and/or chronic form, involves variations in the number and function of lymphocytes, macrophages, and antibodies. This disease features cardiomyocyte abnormalities, which determine regional or global contractile impairment, chamber stiffening, or conduction system disease.^101^ It is a relatively low-incidence pathology for which early diagnosis is hampered by the lack of specific and differential symptoms compared to other heart diseases, being easily confused with different cardiovascular pathologies, *i.e.*, MI.^102,103^ The comparative study was performed in a rat experimental autoimmune myocarditis (EAM) model. Regarding specificity, [^18^F]-CB251 showed superior TSPO uptake in the EAM rat heart compared to [^18^F]-fluoromethyl-PBR28, which did not significantly differ from healthy controls. Results support [^18^F]-CB251 imaging for the noninvasive detection of myocarditis.

The potentiality of [^18^F]-PBR06 (^18^F-*N*-fluoroacetyl-*N-*(2,5-dimethoxybenzyl)-2-phenoxyaniline) as TSPO radiotracer to image atherosclerotic plaques in ApoE-knockout mice provided promising results. A few years later, this ligand and [^11^C]-PBR28 (*N*-acetyl-*N*-(2-^11^C-methoxybenzyl)-2-phenoxy-5-pyridinamine) (Chart 1, Table 2) were used in an *in vitro* and *in vivo* pilot study in humans to evaluate the potential of these compound for the imaging of inflammatory vascular disease.

Despite good uptake on surgical samples *in vitro*, PET studies in patients showed no sign of inflammation *in vivo*, indicating that those two radiotracers failed to prove clinical relevance for imaging inflammatory vessel disease.

[^18^F]-FEDAC (*N*-benzyl-*N*-methyl-2-[7,8-dihydro-7-(2-^18^F-fluoroethyl)-8-oxo-2-phenyl-9*H*-purin-9-yl]acetamide) (Chart 1, Table 2) recently showed, in *in vivo* and *in vitro* studies, the potential to detect atherosclerotic plaques in rabbits and atherosclerotic lesions and high-risk coronary plaques in humans.^104^ In another study, in a rat model of coronary occlusion, [^18^F]-FEDAC demonstrated TSPO is a promising biomarker for imaging mitochondrial dysfunction associated with myocardial ischemia by PET/CT, highlighting the potential of TSPO ligands as tracers for the imaging of ischemic injuries in the heart.^105^ A preliminary evaluation of [^18^F]-FDPA (*N,N*-diethyl-2-(2-(4-^18^F-fluorophenyl)-5,7-dimethylpyrazolo[1,5-*a*]pyrimidin-3-yl) acetamide) (Chart 1, Table 2) for cardiac inflammation imaging was investigated in rats after MI. The stability of uptake in the heart and the fast clearance from the other organs allowed a sufficiently large time window for cardiac imaging. Obtained data highlighted [^18^F]-FDPA as a potential radiotracer for cardiac inflammation.^106^

Second-generation TSPO radioligands possess higher TSPO-specific signals than first-generation radioligands. Conversely, unlike first-generation probes, they suffer from other drawbacks, such as sensitivity to TSPO single-nucleotide polymorphism (SNP) rs6971.^107^ The rs6971 SNP influences the binding of TSPO radioligands in the TSPO gene that determines an amino acid substitution (Ala147Thr). Such polymorphism leads to modifications in the TSPO structure, such as the reduced distance between the second and fifth transmembrane domains, resulting in a lower radioligand affinity. Notably, there are three different human subject categories, namely high-affinity binders (HABs), low-affinity binders (LABs), and mixed-affinity binders (MABs) that are, respectively, homozygous for wild-type TSPO, homozygous for the Ala147Thr TSPO, and heterozygous.^108,109^ Thus, patients with the same TSPO density but different genotypes will provide different PET signals; therefore, TSPO genotyping is required to interpret imaging outcomes. For this reason, a third generation of ligands with low sensitivity toward rs6971 was established (Chart 1, Table 3).

**Table 3:**
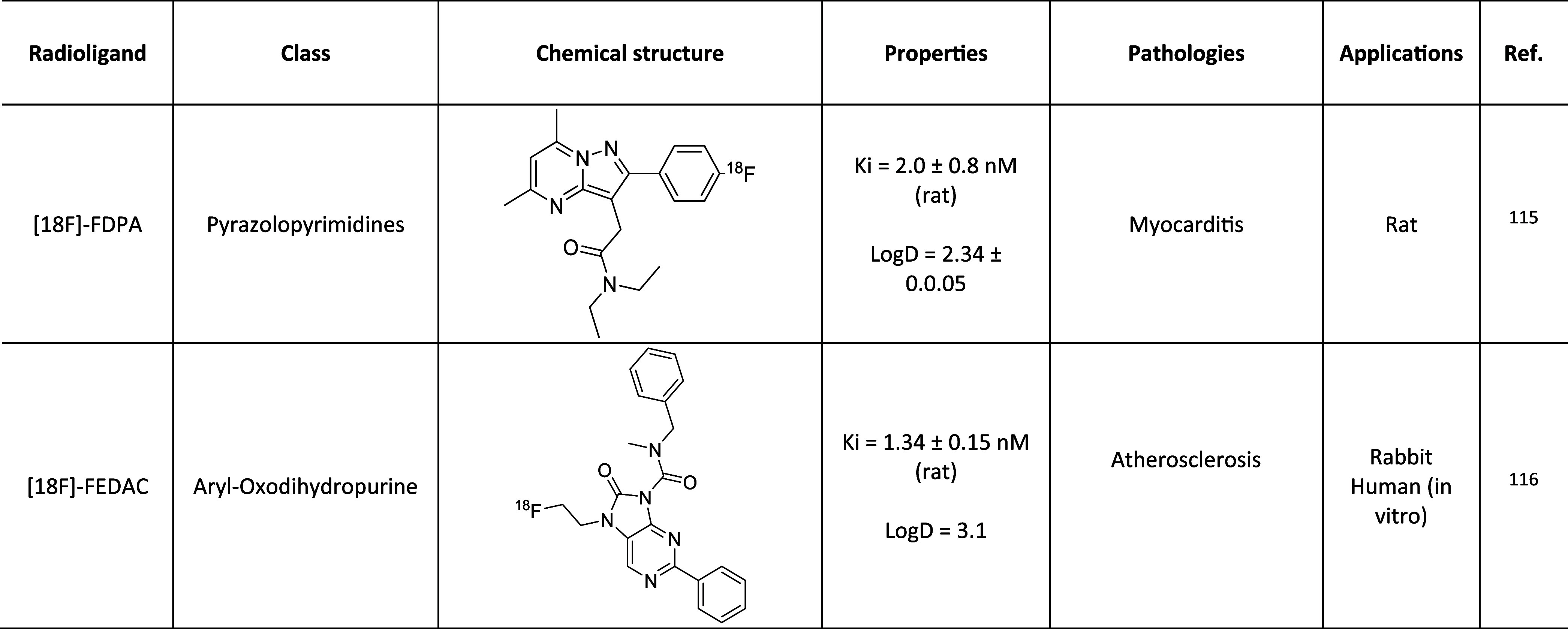
Third-Generation PET Tracers for TSPO

### 3.3 Third-Generation PET Tracers for TSPO

[^18^F]-GE180 (S-*N*,*N*-diethyl-9–2-^18^F-fluoroethyl)-5-methoxy-2,3,4,9-tetrahydro-1*H*-carbazole-4-carboxamide (Chart 1, Table 3)^117^ is a tricyclic-indole compound and has been used in the imaging of atherosclerotic plaque inflammation in a mouse model. [^18^F]-GE180 shares the same high level of TSPO specificity of second-generation ligands and the low sensitivity to SNP of the first-generation class. It showed similar binding characteristics compared to the previously described [^18^F]-FEMPA, displaying uptake in macrophage-rich areas in atherosclerotic lesions and lesion-free vessel walls in mice.^83^ Recently, [^18^F]-LW223 [(*R*)-*N*-(*sec*-butyl)-3-((fluoro-^18^F)methyl)-*N*-methyl-4-phenylquinoline-2-carboxamide] (Chart 1, Table 3) was developed and tested in the detection of macrophage-driven inflammation in a rat MI model.^118^ MI is a life-threatening condition caused by a lack of blood flow and oxygen supply to the heart muscle. It is due mainly to the development of plaques within arterial walls that may occlude the coronary vessels *in situ* or, quite commonly, by vulnerable plaque rupture and distal embolism. The binding of [^18^F]-LW223 to TSPO in the human brain and heart in *in vitro* assays highlighted that it is not susceptible to the rs6971 human genetic polymorphism. In addition, [^18^F]-LW223 detected and quantified the macrophage-driven inflammation in a rat MI model, holding promise in clinical translation for the prognosis of MI.^118^

## 4 TSPO Ligands as Cardioprotective Agents

Several studies have been conducted on TSPO ligands, and their cardioprotective effects have been mainly associated with stabilizing mitochondrial function.

### 4.1 Ro5-4864 (4′-chlorodiazepam)

Ro5-4864 (4′-chlorodiazepam, Figure 1, Table 4) is a TSPO ligand belonging to the class of benzodiazepines in which, basically, a chlorine atom was inserted at the *para* position of the 4-phenyl ring of diazepam, a clinically used benzodiazepine drug.

**Table 4:**
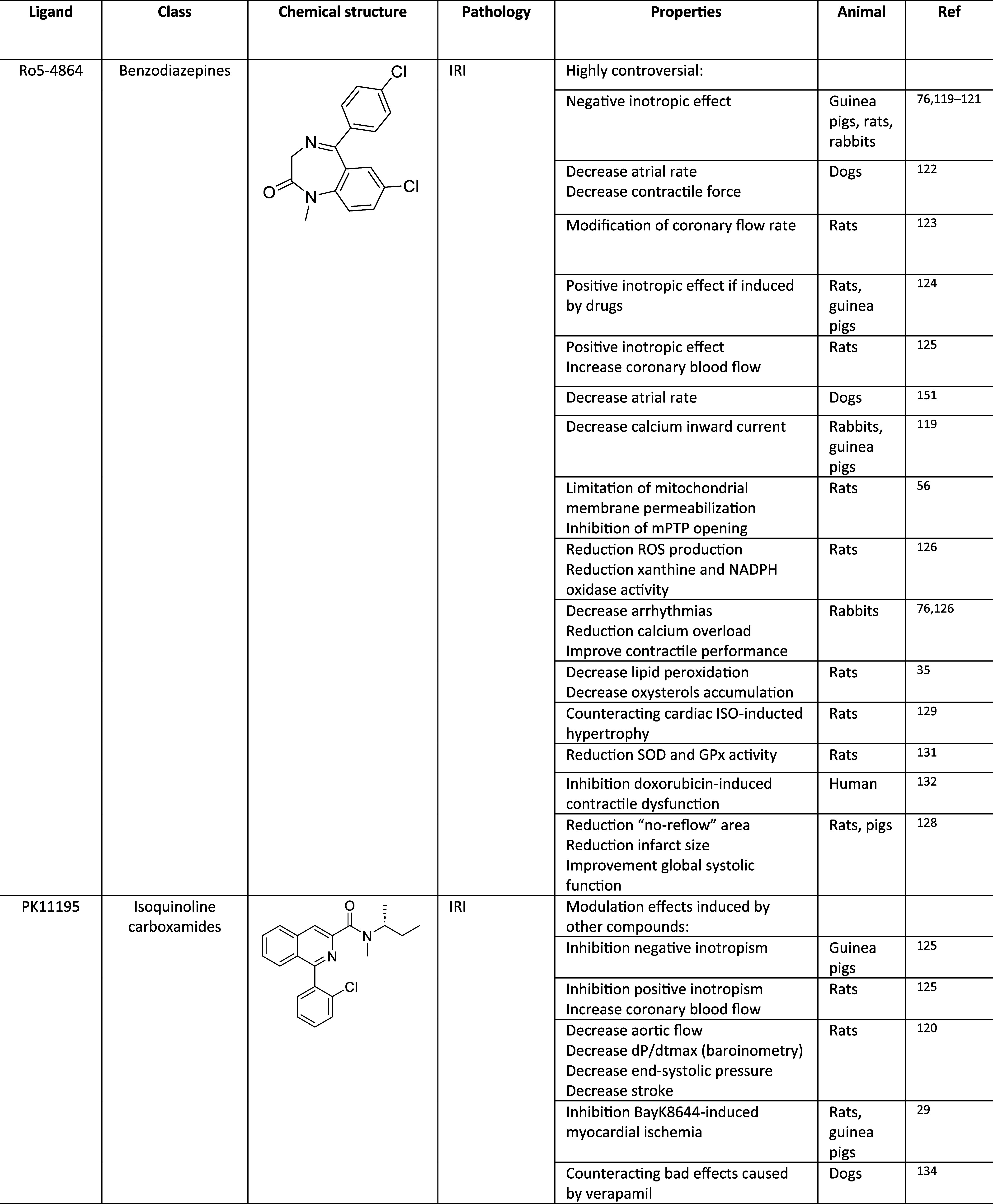

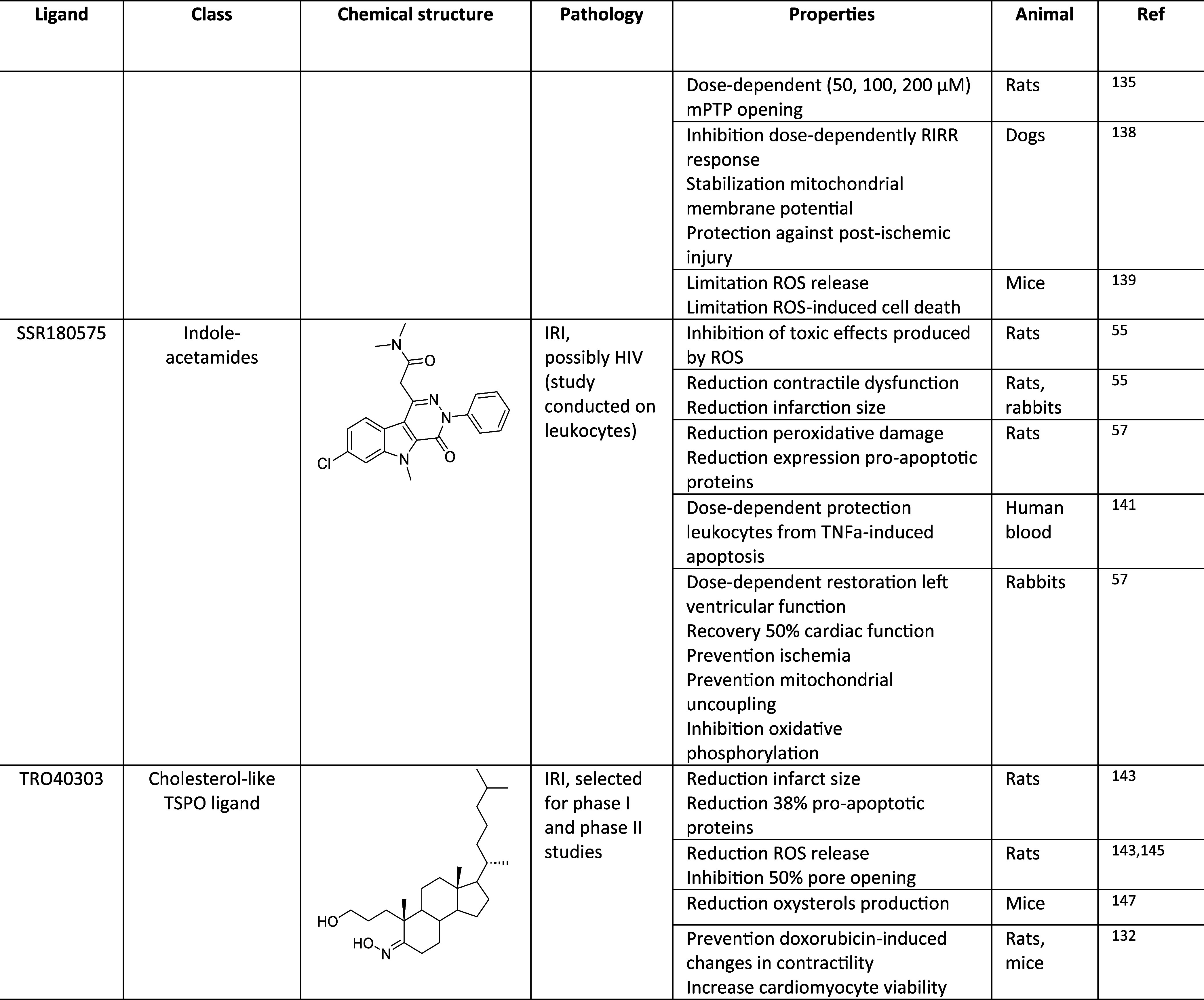
TSPO Cardioprotective Agents

Regarding its role in the physiological activity of the heart (heart rate and contractility), the results reported in the literature have been highly controversial. In a first study, Ro5-4864 showed a negative inotropic effect in several models, for example, in the left and right ventricle papillary muscles of guinea pigs, rats, and rabbits.^76,119−121^ In another study, Saegusa et al. demonstrated that injecting Ro5-4864 into the sinus node of an isolated canine right atrium decreased atrial rate and contractile force at concentrations between 100 and 1000 μg.^122^ Grupp et al. questioned these findings, proving that Ro5-4864 did not show inotropic effects, either positive or negative, but only modified the coronary flow rate by producing a dose-dependent increase in isolated perfused rat hearts.^123^ However, in a following study, Weissman et al.^124^ reported that Ro5-4864 did not act as an inotropic agent by itself but incremented the pro-ischemic inotropic effect induced by a calcium channel activator (BayK8644). On the contrary, Leeuwin et al. demonstrated that Ro5-4864 has a dose-dependent positive inotropic effect and increases coronary blood flow in rat hearts when administered at concentrations greater than 24 μM.^125^ These controversial findings may be due to different doses utilized for the tested ligand or the different animals/species. Leeuwin et al. also demonstrated that the effects mediated by Ro5-4864 were abolished by PK11195.^125^ Also, PET studies highlighted a negative chronotropic action of Ro5-4864 when injected at 200 μg/kg or more,^27^ while a further work demonstrated the role of the ligand in decreasing the calcium inward current during the second phase of the action potential in rabbit and isolated guinea pig cardiomyocytes at concentrations greater than 3 μM.^119^

With respect to pathological conditions, TSPO seems to be mainly involved in IRI. As recently demonstrated by Obame et al., Ro5-4864 was able to reduce cardiac injury and increase recovery during reperfusion following an ischemic event in rats.^56^ This effect is probably associated with inhibition of both Cyt-c release and the activity of AIF. In particular, Ro5-4864 increases the resistance of mitochondria to calcium-induced pore opening by stabilizing the association of the antiapoptotic Bcl-2 with the mitochondrial membrane and hampering the association of the proapoptotic Bax.

To summarize, the cardioprotective effects of Ro5-4864 are related to its ability to limit mitochondrial membrane permeabilization and inhibit mPTP opening.^56^ However, mPTP opening inhibition is not due to a direct action of the ligand on the pore, given that Ro5-4864 did not counteract mPTP opening in isolated mitochondria compared to cyclosporine A, an mPTP opening inhibitor used as reference. Paradoxically, Ro5-4864 induced pore opening at concentrations several orders of magnitude higher than those required to saturate the receptor. In the same study, it has been shown that Ro5-4864 administration resulted in the restoration of mitochondrial respiration and oxidative phosphorylation, which could be associated with the inhibition of mitochondrial Cyt-c release, making it more available for the electron transfer chain.^56^

In 2010, Xiao et al. confirmed these results, indicating that Ro5-4864 reduces ROS production caused by a sudden increase in oxygen during reperfusion and increases the activity of complexes I and III of the mitochondrial electron transport chain while blunting the activity of the ROS generating xanthine and NADPH oxidase.^126^

Ro5-4864 can also decrease the incidence of arrhythmias and reduce calcium overload. In addition, if administered at reperfusion, it protects against postischemic arrhythmias following reperfusion in rabbit hearts; if administered before ischemia, it improves the recovery of the post-IR contractile performance.^76,126^ In a preclinical study conducted on pigs, Ro5-4864 intracoronary administration at the onset of reperfusion, after 60 min of coronary occlusion, improved faster ST-segment elevation resolution without hemodynamic complications and reduced microvascular damage, but it did not significantly reduce infarct size.^127^

As mitochondrial membrane fluidity appears to be impaired after cardiac IR, Paradis et al. investigated the causes of this membrane alteration on mitochondria isolated from rat hearts subjected to a half-hour ischemia followed by a quarter-hour reperfusion; the administration of Ro5-4864 appeared to ameliorate this condition.^35^ Specifically, membrane fluidity was measured by evaluating the change in steady-state fluorescence anisotropy of two fluorescent probes, namely 1,6-diphenyl-1,3,5-hexatriene (DPH) and hematoporphyrin IX (HP), that bound, respectively, to hydrophobic lipid regions and protein sites in mitochondrial membranes. The results indicated that cholesterol accumulation in the mitochondrial matrix in the post-IR setting indirectly affected membrane fluidity at lipid regions by promoting lipid peroxidation, which was prevented by the administration of Ro5-4864. Cholesterol accumulation, established during reperfusion, remains one of the major problems related to the cardiovascular system, as it leads to atherosclerosis, arterial stenosis, thrombosis, and myocardial ischemia. In this respect, Paradis et al. also showed that Ro5-4864 strongly inhibited the accumulation of cholesterol and cholesterol-derived oxidation compounds (oxysterols) during reperfusion, improved respiration parameters, and decreased the sensitivity of mPTP opening.^35^ Collectively, these data demonstrate that one of the main cardioprotective effects exerted by Ro5-4864 is the limitation of dangerous effects exerted by cholesterol accumulation and lipid peroxidation to limit mitochondrial membrane derangements and functional impairments.

In a study conducted in 2016, the authors^128^ compared the effects of intracoronary Ro5-4864 (2 μM) administered to pigs or rats just prior to or immediately after reperfusion, respectively. The treatment causes a reduction of the “no-reflow” area and guarantees long-term positive effects, such as a reduction in infarct size and improvement in global systolic function in rats. In large animals (pigs), closer to the human physiology, Ro5-4864 caused more or less the same effects with the addition of more rapid resolution of ST-segment elevation.

Another study conducted in 2010 concerning oxidative stress proved that Ro5-4864 could counteract cardiac hypertrophy in rats induced by isoproterenol (ISO),^129^ a nonselective β-adrenergic agonist associated with an increase in oxidative stress, fibrosis, and hypertrophy. ISO binding to β-adrenergic receptors causes positive inotropic and chronotropic effects; specifically, it causes both peripheral vasodilatation and cardiac hypoxia, resulting in relative ischemia and calcium overload leading to excessive ROS production, oxidative stress, infarct-like cardiomyocyte necrosis, and myocardial fibrosis. The results showed that Ro5-4864 decreased myocyte hypertrophy, fibrosis, and necrosis, which were previously increased by the high levels of ROS. Concerning myosin heavy chain (MHC) isoform expression in murine models, in physiological conditions the β-isoform is less expressed than the α-isoform, but in the case of cardiovascular problems the former is upregulated; in fact, β-MHC is considered a marker of hypertrophy. Administration of Ro5-4864 resulted in the downregulation of β-MHC expression and the reduction of left ventricular size. Important data emerged from this study regarding Ro5-4864 doses. Indeed, while the decrease in isoprenaline-induced production of thiobarbituric acid reactive substances (TBARs) did not change with increasing doses, different data were found on reducing two endogenous antioxidants, glutathione peroxidase (GPx) and superoxide dismutase (SOD). In fact, Ro5-4864 at 0.1 mg/kg inhibited the decrease in both antioxidants, but when the doses were increased to 0.5 mg/kg the effect was maintained only toward the SOD.^129^ The explanation for this trend is not clear from this study, but Ro5-4864 has already demonstrated to have such paradoxical effects in terms of concentrations. Indeed, from a previous *in vitro* study conducted by Kenyon et al., it emerged that nanomolar concentrations of Ro5-4864 caused aldosterone release, but this effect is not retained by increasing concentrations to a micromolar range. This could be due to inhibitory effects involving enzyme competition.^130^ Clearly the cardioprotective effects of the TSPO ligand are complex and need more focused studies.

In a more recent study, the possible cardioprotective effects of Ro5-4864, alone or in the presence of the NO synthase (NOS) inhibitor N(ω)-nitro-l-arginine methyl ester (L-NAME), were evaluated in a model of ISO-induced rat MI.^131^ Lower levels of circulating and myocardial markers of ischemic injuries were measured in the Ro5-4864-treated group, which was partially prevented by L-NAME, thus suggesting that NO should be an important mediator of the TSPO ligand action.^131^

In another work, the role of Ro5-4864 against doxorubicin-induced cardiac damage has been evaluated. Doxorubicin is an antineoplastic antibiotic of the anthracycline family with a broad antitumor spectrum. However, it causes contractile dysfunction and the development of cardiomyopathies related to mitochondrial dysfunction, proved by the fact that the mitochondrial respiratory chain is the primary source of ROS during the treatment. In this context, the effects of Ro5-4864 have been studied on adult isolated-paced cardiomyocytes. The results evidenced that Ro5-4864 inhibits the doxorubicin-induced contractile dysfunction, which initially manifests as acute myocardial injury and then advances to a chronic congestive HF by augmenting ROS production and mPTP opening.^132^

### 4.2 PK11195

PK11195 (Figure 1, Table 4) is a TSPO ligand belonging to the class of isoquinoline carboxamides.^133^ It should be remembered that PK11195 influences neither heart contractility nor coronary flow rate by itself. Still, it modulated Ro5-4864 effects, even if the two ligands display a similar affinity for TSPO. In fact, PK11195 was shown to inhibit the effects produced by Ro5-4864 on the heart, including the negative inotropism on papillary muscle from the right and left ventricles of guinea pigs and the dose-dependent positive inotropism together with the increase of coronary blood flow in rat hearts.^125^ Nevertheless, PK11195 resulted in diminishing aortic flow, d*P*/d*t*_max_ (baroinometry), end-systolic pressure, and stroke work more than Ro5-4864;^120^ this may be due to the interactions with the target that are influenced by the nature of the ligand in terms of enthalpy and entropy, which are the basis of the binding equilibrium.^133^

Cytosolic calcium concentrations play a crucial role in ischemic processes. Therefore, it is thought that the interaction of TSPO with L-type voltage-dependent calcium channels located on the plasma membrane may contribute to the development of this pathological condition.

As previously explained in section 4.1, Ro5-4864 can increase BayK8644-induced myocardial ischemia; in contrast, PK11195 has been shown to inhibit it.^29^ Both Ro5-4864 and PK111195 could also have a direct action on calcium channels, in addition to that via TSPO. In fact, in a study conducted in dogs treated with verapamil, an antihypertensive calcium antagonist that induced heart toxicity, PK11195 proved to counteract some effects caused by the drug. For example, PK11195 could restore sinus activity lost during verapamil treatment but could not prevent or cure hemodynamic changes.^134^

In a study conducted in 2006,^135^ the ability of PK11195 to promote mPTP opening, leading consequently to Cyt-c release and mitochondrial uncoupling, was investigated. The results showed that PK11195 causes dose-dependent (50, 100, and 200 μM) mPTP opening. In general, a calcium concentration above 100 μM is required to cause pore opening,^136^ but the results of this study showed that PK11195-induced opening does not require calcium, consistent with the fact that it can interact directly with voltage-dependent calcium channels located on the plasma membrane.^137^ This study also revealed that CsA prevented PK11195-induced opening, inhibiting pro-apoptotic protein release.

Regarding oxidative stress, PK11195, at concentrations from 5 to 25 mg/kg, resulted in the dose-dependent inhibition of the RIRR response, stabilizing mitochondrial membrane potential and protecting against postischemic injury and early and delayed ventricular arrhythmias induced in dogs by 20 min of ischemia.^138^

In a more recent study conducted in 2020, PK11195 was shown to have a unique mechanism to limit the RIRR phenomenon in rabbits. Indeed, when administered at the moment of reperfusion, PK11195 limited ROS release and ROS-induced cell death.^139^ In more detail, rabbit ventricular myocytes were subjected to 20 min of ischemia and then to 3 h of reperfusions in both the absence and the presence of 50 μM PK11195 treatments, the first after 15 min, the second after 1 h, and the third after 3 h from reperfusion. Myocyte death was assessed by lactate dehydrogenase (LDH) assay, while other effects, such as changes in calcium concentration, membrane potential, and ROS release, were examined by confocal microscopy combined with fluorescent indicators. An important finding emerged from the results; it was shown that PK11195, administered at the onset of reperfusion, normalized succinate oxidation and glutamate utilization. However, when administered earlier during ischemia, it did not exert cardioprotective effects. These findings highlighted the importance of the timing of PK11195 administration during myocardial ischemia.^139^

### 4.3 SSR180575

SSR180575 (7-chloro-*N,N*,5-trimethyl-4-oxo-3-phenyl-3,5-dihydro-4*H*-pyridazino[4,5-*b*]indole-1-acetamide, Table 4) is a TSPO ligand that features both neuroprotective and cardioprotective effects.^57^ As mitochondria not only represent an energy source but also regulate the cell life–death cycle in case of pathological conditions, ROS production represents one of the major problems in IRI; in particular, H_2_O_2_ causes a massive reduction of mitochondrial membrane potential, inhibition of oxidative phosphorylation, and release of pro-apoptotic proteins such as caspase 3. In several *in vitro* and *in vivo* models of cardiac IRI, SSR180575 prevented the ROS-dependent decrease of mitochondrial membrane potential, reduced oxidative phosphorylation capacities, Cyt-c release, caspase 3 activation, and DNA fragmentation.^55^ In more detail, administering SSR180575 in perfused rat hearts (100 nM to 1 μM) or by oral pretreatment (3–30 mg/kg), reduced contractile dysfunction caused by reperfusion. Furthermore, a marked reduction in infarction size was noted in both isolated rabbit hearts and anaesthetized rats undergoing left coronary artery occlusion followed by reperfusion; this is probably related to the coexistence of necrosis and apoptosis during ischemia, which diminishes in case of treatment with SSR180575.^140^ Still, administration of SSR180575 both preventively and therapeutically restored left ventricular function in a dose-dependent manner following an ischemic event in a model of rabbit hearts. SSR180575 also improved recovery of cardiac function by 50% and prevented ischemia-induced reperfusion, the primary cause of cardiac damage.^55^ Regarding oxidative stress, it was noted that SSR180575 prevented mitochondrial uncoupling and inhibition of oxidative phosphorylation in treated mitochondria subjected to H_2_O_2_. Therefore, it is thought that it may act directly on mitochondria involved in calcium homeostasis, one of the critical points of ischemic damage.^57^

Another study has determined TSPO’s role in signaling pathways that lead to cell death. In particular, the study focused on renal dysfunction in a rat IR model. Similar to what was observed for the heart, ischemia followed by reperfusion caused both apoptosis and tubular necrosis in kidneys due to increased production of ROS, thus determining peroxidative damage, high expression of the proapoptotic protein Bax, low expression of the antiapoptotic protein Bcl-2, and caspase 3 release. Treatment with SSR180575 significantly reduced these damaging effects.^57^ Another study on apoptosis was conducted in 2010 on polymorphonuclear leukocytes (PMNs), as TSPO is highly expressed in blood cells. Specifically, administration of SSR180575 protects leukocytes from TNFα-induced apoptosis in a dose-dependent manner, thus leading to the conviction that targeting TSPO could represent a potential strategy for the treatment of pathologies characterized by an increased blood cell apoptosis, such as HIV (human immunodeficiency virus).^141^

### 4.4 TRO40303

TRO40303, namely 3,5-seco-4-nor-cholestan-5-one oxime-3-ol (Table 4), is a cholesterol-like TSPO ligand that binds specifically to the cholesterol site of TSPO; it was initially identified as a neuroprotective agent^142^ but was later investigated in more detail for its roles in cardioprotection, as [^14^C]TRO40303 accumulates rapidly in rats’ hearts after a single intravenous administration (2 mg/kg).^143^ In the same study, rats were subjected to 35 min of ischemia and then reperfused for 24 h. Administration of TRO40303 (2.5 mg/kg) immediately before reperfusion reduced infarct size by 38%, and this was associated with a reduction in pro-apoptotic proteins, especially AIF, but not Cyt-c release, which is a major cause of cell death.^144^ TRO40303 showed no effect on calcium retention but still caused a delay in mPTP opening in rats subjected to 2 h of ischemia followed by 2 h of reperfusion and inhibited pore opening in cardiomyocytes of neonatal rats treated with H_2_O_2_. It is therefore thought that the delayed mPTP opening is not due to an action on calcium retention but to a reduction in ROS production. Comparing the effects of TRO40303 with those of CsA on pore opening and oxidative stress, it was noted that TRO-dependent effects occur earlier than those caused by CsA and, in particular, mPTP opening inhibition promoted by CsA coincides with its effects on ROS production and calcium overload. In addition, it was found that effects mediated by both CsA and TRO40303 on mitochondrial and cytosolic calcium concentration increases are similar, demonstrating that Ca^2+^ increase is a secondary and a pore-opening-dependent effect. By subjecting cardiomyocytes to H_2_O_2_-induced oxidative stress with subsequent ROS production, different effects were observed for TRO40303 and CsA. Specifically, TRO40303 drastically reduced ROS release, while CsA caused a much smaller ROS reduction; however, both TRO40303 and CsA inhibited pore opening by 50%. This effect could be explained by the fact that while TRO40303 inhibits mPTP opening by reducing the production of ROS, CsA acts by binding and inhibiting CypD, a protein that acts as a gate within the pore, decreasing the release of Cyt-c that causes programmed cell death.^143,145^ It is worth mentioning that TRO40303 is a TSPO ligand that binds to the cholesterol site, unlike the other ligands discussed above. Therefore, it could also exert its action by binding directly to specific components related to TSPO, such as VDAC, promoting its interaction with a hexokinase that leads to the maintenance of the required ATP concentrations and augmenting glycolysis.^146^ Furthermore, TRO40303 also reduced the oxysterol production leading to a minor cholesterol accumulation into the mitochondrial membrane during reperfusion, diminishing the related injury and the complications due to the presence of comorbidities such as dyslipidemia and hypercholesterolemia.^147^ Still related to ROS production, TRO40303 prevented the doxorubicin-induced changes in contractility and augmented cardiomyocyte viability, as previously reported for Ro5-4864.^132^ It is of great importance that TRO40303 was selected as a cardioprotective agent for a randomized phase I study carried out double-blind. Specifically, the study was based on the treatment of healthy male subjects, together with postmenopausal and hysterectomized women, with TRO40303 administered intravenously from 0.5–13 mg/kg with a flow rate of 0.04–35 mL/min. The results showed that the doses were tolerated without causing any major adverse effects, clarifying that the active dose is 6 mg/kg.^148^ As previously mentioned, timely revascularization following an ischemic event is currently the only accepted therapy in cases of MI, despite causing the so-called reperfusion injury. For this reason, TRO40303 was selected as a cardioprotective agent for a phase II study (MITOCARE), a double-blind trial in which its safety and efficacy in limiting reperfusion injury were evaluated. In particular, reperfused patients treated for acute ST-elevation myocardial infarction (STEMI) with percutaneous coronary intervention (PCI) or thrombolysis were treated with TRO40303 or a placebo in addition to their current standard cardiac drugs. Efficacy was assessed primarily by measuring infarct size expressed as area under the curve (AUC) for plasma creatine kinase (CK) and troponin I over 3 days and secondarily by measuring infarct size normalized to the myocardium at risk using cardiac magnetic resonance (CMR) together with the evaluation of left ventricular function, echocardiography, ST-segment decrease, microvascular obstruction, and extension of the infarct after PCI.^149^ Unfortunately, data showed no major differences in the reduction of reperfusion damage and infarction between TRO40303-treated and placebo-treated subjects. These results raised a more provocative question of whether reperfusion injury occurs in humans, which calls for further and more in-depth studies.^150^

## 5 Future Perspectives and Conclusions

Cardiovascular diseases are the leading causes of morbidity, disability, and death in Europe and worldwide. In the last two years, SARS-CoV2 (severe acute respiratory syndrome coronavirus 2) infection has been an aggravating factor, enhancing cardiovascular frailty due to inflammation and stress. The current evidence suggests that TSPO ligands are promising cardioprotective candidates, primarily acting at the level of mitochondria to contrast oxidative stress and optimize the flux and fate of cholesterol away from the accumulation of toxic cholesterol catabolites in mitochondria. In this way, TSPO may contribute to the modulation of inflammation. However, the exact molecular mechanisms of TSPO action in the heart remain largely debated and unclarified, together with the clinical outcomes of TSPO-based drug agents. We reviewed the characteristics of several pharmacological agents in detail. So far, studies have been controversial on the outcomes of TSPO-based drugs on cardiac contractility and perfusion under physiological conditions, which may be partly attributed to the variable models or drug dosage adopted in different studies. More consistent findings support a beneficial role in alleviating IRI, in which a variety of targets have been proposed, including apoptosis-inducing factor (AIF), mitochondrial respiration and reactive oxygen species (ROS) production, calcium load (affecting cardiac rhythm), and lipid peroxidation (affecting membrane stability). However, the reduction of infarct size was only observed sporadically in specific models and selected circumstances.

In principle, molecular imaging by PET represents an ideal tool to characterize the *in vivo* sites of action of TSPO. PET imaging of radiolabeled TSPO ligands has been more traditionally investigated in the brain, where an elevation in TSPO binding reflects the activation of microglial cells and is considered a biomarker of neuroinflammation. Building on this inheritance, the imaging paradigm has been translated into the cardiovascular area with an initial focus on the inflammatory states accompanying CVDs, namely atherosclerotic plaque vulnerability, vasculitis, and myocarditis. Three generations of TSPO-targeted PET tracers have been synthesized and tested. First-generation tracers proved to be insufficiently sensitive to stratify plaque vulnerability but may still prove useful in diagnosing more severe inflammation in vasculitis. Second-generation probes were able to capture myocarditis or cardiac inflammation following MI; some of them hold promise in the diagnosis of inflamed plaques in animals, and one ([^18^F]-FEDAC) also holds promise in humans. However, their binding and imaging signal is influenced by gene polymorphisms, hampering the interpretation of images unless genotyping is contextually performed. Third-generation probes appear to be less sensitive to genetic variability, showing a similar capacity to detect atherosclerotic plaque inflammation compared to second-generation radioligands as well as promising sensitivity to detect macrophage-driven inflammation in MI.

In conclusion, the study of TSPO in the heart has opened new diagnostic and therapeutic leads to address CDs. A better mechanistic understanding of such a multisite-acting protein requires systematic studies comparing disease models, doses, responses, and mechanisms in a consistent manner. TSPO-PET imaging has been so far used to seek inflammation; however, in light of the above-summarized mechanisms and their known involvement in different phases of CD damage, repair, and remodeling, it would seem appropriate to image the heart and vessels along disease phases, from insult to dysfunction to symptomatic disease. In fact, it is tempting to speculate that TSPO binding may signal different mechanisms in different phases. The development of organelle-selective PET tracers would greatly enhance the potential of molecular imaging to contribute to understanding the described complexity and contradictions.

In this respect, the authors believe that the present overview on the advantages and limitations of TSPO ligands will help to design and develop new therapeutic and/or diagnostic tools with better efficacy, which will lead to unraveling the fundamental mechanisms and providing solutions to still unanswered questions in CDs.

## Abbreviations Used

AIF: apoptosis-inducing factor
ANT: adenine nucleotide translocase
AUC: area under the curve
CDs: cardiac disorders
OHC: hydroxycholesterol
CK: creatine kinase
CMR: cardiac magnetic resonance
CsA: cyclosporin A
CT: computed tomography
CTR: cervico-thoracic ratio
CVDs: cardiovascular diseases
CypD: cyclophilin D
CYP11A1: cytochrome P-450 family 11 subfamily A member 1
Cyt-c: cytochrome-c
DPH: 1,6-diphenyl-1,3,5- hexatriene
EAM: experimental autoimmune myocarditis
GPx: glutathione peroxidase
HABs: high-affinity binders
HF: heart failure
HIV: human immunodeficiency virus
HP: hematoporphyrin IX
IMAC: inner membrane anion channel
IMM: inner mitochondrial membrane
IR: ischemia reperfusion
IRI: ischemia-reperfusion injury
ISO: isoproterenol
Laboratories: low-affinity binders
LDH: lactate dehydrogenase
L-NAME: N(ω)-nitro-l-arginine methyl ester
LVV: large vessel vasculitis
MABs: mixed affinity binders
MHC: myosin heavy chain
MI: myocardial infarction
mPTP: mitochondrial permeability transition pore
NIM811: *N*-methyl-4-isoleucine-CsA
Debio-025: D-3- MeAla-4-EtVal-CsA
NO: nitric oxide
NOS: NO synthase
OMM: outer mitochondrial membrane
PBR: peripheral benzodiazepine receptor
PCI: percutaneous coronary intervention
PET: Positron Emission Tomography
PMNs: polymorphonuclear leukocytes
PPIase: peptidyl-prolyl *cis–trans* isomerase
RIRR: ROS-induced ROS release
ROS: reactive oxygen species
SARS-CoV2: severe acute respiratory syndrome coronavirus 2
SfA: sangliferin A
SNP: single nucleotide polymorphism
SOD: superoxide dismutase
STEMI: ST-elevation myocardial infarction
TBARs: thiobarbituric acid reactive substances
TSPO: translocator protein (18 kDa)
VDAC: voltage-dependent anion channel
β-MHC: β-myosin heavy chain

## References

[ref1] Benjamin E. J.; Muntner P.; Alonso A.; Bittencourt M. S.; Callaway C. W.; Carson A. P.; Chamberlain A. M.; Chang A. R.; Cheng S.; Das S. R.; Delling F. N.; Djousse L.; Elkind M. S. V.; Ferguson J. F.; Fornage M.; Jordan L. C.; Khan S. S.; Kissela B. M.; Knutson K. L.; Kwan T. W.; Lackland D. T.; Lewis T. T.; Lichtman J. H.; Longenecker C. T.; Loop M. S.; Lutsey P. L.; Martin S. S.; Matsushita K.; Moran A. E.; Mussolino M. E.; O’Flaherty M.; Pandey A.; Perak A. M.; Rosamond W. D.; Roth G. A.; Sampson U. K. A.; Satou G. M.; Schroeder E. B.; Shah S. H.; Spartano N. L.; Stokes A.; Tirschwell D. L.; Tsao C. W.; Turakhia M. P.; VanWagner L. B.; Wilkins J. T.; Wong S. S.; Virani S. S. Heart Disease and Stroke Statistics-2019 Update: A Report From the American Heart Association. Circulation 2019, 139 (10), e56–e528. 10.1161/CIR.0000000000000659. 30700139

[ref2] Guilarte T. R. TSPO in Diverse CNS Pathologies and Psychiatric Disease: A Critical Review and a Way Forward. Pharmacol Ther 2019, 194, 44–58. 10.1016/j.pharmthera.2018.09.003. 30189290 PMC6348013

[ref3] Mcenery M. W.; Snowman A. M.; Trifiletti R. R.; Snyder S. H. Isolation of the Mitochondrial Benzodiazepine Receptor: Association with the Voltage-Dependent Anion Channel and the Adenine Nucleotide Carrier. Proc. Natl. Acad. Sci. U. S. A. 1992, 89 (8), 3170–3174. 10.1073/pnas.89.8.3170. 1373486 PMC48827

[ref4] Kokoszka J. E.; Waymire K. G.; Levy S. E.; Sligh J. E.; Cai J.; Jones D. P.; Macgregor G. R.; Wallace D. C. The ADP/ATP Translocator Is Not Essential for the Mitochondrial Permeability Transition Pore. Nature 2004, 427 (6973), 461–465. 10.1038/nature02229. 14749836 PMC3049806

[ref5] Baines C. P.; Kaiser R. A.; Sheiko T.; Craigen W. J.; Molkentin J. D. Voltage-Dependent Anion Channels Are Dispensable for Mitochondrial-Dependent Cell Death NIH Public Access Author Manuscript. Nat. Cell Biol. 2007, 9 (5), 550–555. 10.1038/ncb1575. 17417626 PMC2680246

[ref6] Cheng E. H. Y.; Sheiko T. V.; Fisher J. K.; Craigen W. J.; Korsmeyer S. J. VDAC2 Inhibits BAK Activation and Mitochondrial Apoptosis. Science 2003, 301 (5632), 513–517. 10.1126/science.1083995. 12881569

[ref7] Šileikyte J.; Blachly-Dyson E.; Sewell R.; Carpi A.; Menabò R.; Di Lisa F.; Ricchelli F.; Bernardi P.; Forte M. Regulation of the Mitochondrial Permeability Transition Pore by the Outer Membrane Does Not Involve the Peripheral Benzodiazepine Receptor (Translocator Protein of 18 KDa (TSPO)). J. Biol. Chem. 2014, 289 (20), 13769–13781. 10.1074/jbc.M114.549634. 24692541 PMC4022851

[ref8] Papadopoulos V.; Baraldi M.; Guilarte T. R.; Knudsen T. B.; Lacapère J. J.; Lindemann P.; Norenberg M. D.; Nutt D.; Weizman A.; Zhang M. R.; Gavish M. Translocator Protein (18 kDa): New Nomenclature for the Peripheral-Type Benzodiazepine Receptor Based on Its Structure and Molecular Function. Trends Pharmacol. Sci. 2006, 27 (8), 402–409. 10.1016/j.tips.2006.06.005. 16822554

[ref9] Fan J.; Lindemann P.; Feuilloley M. G. J.; Papadopoulos V. Structural and Functional Evolution of the Translocator Protein (18 KDa). Curr. Mol. Med. 2012, 12 (4), 369–386. 10.2174/1566524011207040369. 22364126

[ref10] Costa B.; Da Pozzo E.; Martini C. Translocator Protein and Steroidogenesis. Biochem. J. 2018, 475 (5), 901–904. 10.1042/BCJ20170766. 29511094

[ref11] Costa B.; Da Pozzo E.; Giacomelli C.; Barresi E.; Taliani S.; Da Settimo; Martini C. TSPO Ligand Residence Time: A New Parameter to Predict Compound Neurosteroidogenic Efficacy. Sci. Rep 2016, 6, 18164 10.1038/srep18164. 26750656 PMC4707509

[ref12] Ritsner M.; Modai I.; Gibel A.; Leschiner S.; Silver H.; Tsinovoy G.; Weizman A.; Gavish M. Decreased Platelet Peripheral-Type Benzodiazepine Receptors in Persistently Violent Schizophrenia Patients. J. Psychiatr Res. 2003, 37 (6), 549–556. 10.1016/S0022-3956(03)00055-4. 14563387

[ref13] O’Hara M. F.; Nibbio B. J.; Craig R. C.; Nemeth K. R.; Charlap J. H.; Knudsen T. B. Mitochondrial Benzodiazepine Receptors Regulate Oxygen Homeostasis in the Early Mouse Embryo. Reproductive Toxicology 2003, 17 (4), 365–375. 10.1016/S0890-6238(03)00035-2. 12849846

[ref14] Larcher J. C.; Vayssiere J. L.; Le Marquer F. J.; Cordeau L. R.; Keane P. E.; Bachy A.; Gross F.; Croizat B. P. Effects of Peripheral Benzodiazepines upon the O2 Consumption of Neuroblastoma Cells. Eur. J. Pharmacol. 1989, 161 (2–3), 197–202. 10.1016/0014-2999(89)90843-1. 2542045

[ref15] Hauet T.; Yao Z. X.; Bose H. S.; Wall C. T.; Han Z.; Li W.; Hales D. B.; Miller W. L.; Culty M.; Papadopoulos V. Peripheral-Type Benzodiazepine Receptor-Mediated Action of Steroidogenic Acute Regulatory Protein on Cholesterol Entry into Leydig Cell Mitochondria. Mol. Endocrinol. 2005, 19 (2), 540–554. 10.1210/me.2004-0307. 15498831

[ref16] Ostuni M. A.; Marazova K.; Peranzi G.; Vidic B.; Papadopoulos V.; Ducroc R.; Lacapere J. J. Functional Characterization and Expression of PBR in Rat Gastric Mucosa: Stimulation of Chloride Secretion by PBR Ligands. Am. J. Physiol. Gastrointest. Liver Physiol. 2004, 286 (6), G1069–G1080. 10.1152/ajpgi.00290.2003. 14726306

[ref17] Veenman L.; Gavish M. The Peripheral-Type Benzodiazepine Receptor and the Cardiovascular System. Implications for Drug Development. Pharmacol Ther 2006, 110 (3), 503–524. 10.1016/j.pharmthera.2005.09.007. 16337685

[ref18] O’Hara M. F.; Charlap J. H.; Craig R. C.; Knudsen T. B. Mitochondrial Transduction of Ocular Teratogenesis during Methylmercury Exposure. Teratology 2002, 65 (3), 131–144. 10.1002/tera.10028. 11877777

[ref19] Han Z.; Slack R. S.; Li W.; Papadopoulos V. Expression of Peripheral Benzodiazepine Receptor (PBR) in Human Tumors: Relationship to Breast, Colorectal, and Prostate Tumor Progression. J. Recept Signal Transduct Res. 2003, 23 (2–3), 225–238. 10.1081/RRS-120025210. 14626449

[ref20] Setiawan E.; Wilson A. A.; Mizrahi R.; Rusjan P. M.; Miler L.; Rajkowska G.; Suridjan I.; Kennedy J. L.; Rekkas P. V.; Houle S.; Meyer J. H. Role of Translocator Protein Density, a Marker of Neuroinflammation, in the Brain during Major Depressive Episodes. JAMA Psychiatry 2015, 72 (3), 268–275. 10.1001/jamapsychiatry.2014.2427. 25629589 PMC4836849

[ref21] Barresi E.; Robello M.; Costa B.; Da Pozzo E.; Baglini E.; Salerno S.; Da Settimo F.; Martini C.; Taliani S. An Update into the Medicinal Chemistry of Translocator Protein (TSPO) Ligands. Eur. J. Med. Chem. 2021, 209, 112924 10.1016/j.ejmech.2020.112924. 33081988

[ref22] Veenman L.; Levin E.; Weisinger G.; Leschiner S.; Spanier I.; Snyder S. H.; Weizman A.; Gavish M. Peripheral-Type Benzodiazepine Receptor Density and in Vitro Tumorigenicity of Glioma Cell Lines. Biochem. Pharmacol. 2004, 68 (4), 689–698. 10.1016/j.bcp.2004.05.011. 15276076

[ref23] Salvetti F.; Chelli B.; Gesi M.; Pellegrini A.; Giannaccini G.; Lucacchini A.; Martini C. Effect of Noise Exposure on Rat Cardiac Peripheral Benzodiazepine Receptors. Life Sci. 2000, 66 (13), 1165–1175. 10.1016/S0024-3205(00)00422-7. 10737412

[ref24] Davies L. P.; Huston V. Peripheral Benzodiazepine Binding Sites in Heart and Their Interaction with Dipyridamole. Eur. J. Pharmacol. 1981, 73 (2–3), 209–211. 10.1016/0014-2999(81)90092-3. 6273185

[ref25] Gehlert D. R.; Yamamura H. I.; Wamsley J. K. Autoradiographic Localization of “Peripheral-Type” Benzodiazepine Binding Sites in the Rat Brain, Heart and Kidney. Naunyn Schmiedebergs Arch Pharmacol 1985, 328 (4), 454–460. 10.1007/BF00692915. 2986017

[ref26] Basile A. S.; Weissman B. A.; Skolnick P. Maximal Electroshock Increases the Density of [3H]Ro 5–4864 Binding to Mouse Cerebral Cortex. Brain Res. Bull. 1987, 19 (1), 1–7. 10.1016/0361-9230(87)90158-4. 2820550

[ref27] Surinkaew S.; Chattipakorn S.; Chattipakorn N. Roles of Mitochondrial Benzodiazepine Receptor in the Heart. Can. J. Cardiol 2011, 27 (2), 262.e3–262.e13. 10.1016/j.cjca.2010.12.023. 21459278

[ref28] Morin D.; Musman J.; Pons S.; Berdeaux A.; Ghaleh B. Mitochondrial Translocator Protein (TSPO): From Physiology to Cardioprotection. Biochem. Pharmacol. 2016, 105, 1–13. 10.1016/j.bcp.2015.12.003. 26688086

[ref29] Bolger G. T.; Abraham S.; Oz N.; Weissman B. A. Interactions between Peripheral-Type Benzodiazepine Receptor Ligands and an Activator of Voltage-Operated Calcium Channels. Can. J. Physiol. Pharmacol. 1990, 68 (1), 40–45. 10.1139/y90-005. 1691678

[ref30] Mangoni M. E.; Marger L.; Nargeot J. If Current Inhibition: Cellular Basis and Physiology. Adv. Cardiol 2006, 43, 17–30. 10.1159/000095403. 16936469

[ref31] Bender A. S.; Hertz L. Pharmacological Evidence That the Non-Neuronal Diazepam Binding Site in Primary Cultures of Glial Cells Is Associated with a Calcium Channel. Eur. J. Pharmacol. 1985, 110 (2), 287–288. 10.1016/0014-2999(85)90226-2. 2580725

[ref32] Iuliano L. Pathways of Cholesterol Oxidation via Non-Enzymatic Mechanisms. Chem. Phys. Lipids 2011, 164 (6), 457–468. 10.1016/j.chemphyslip.2011.06.006. 21703250

[ref33] Brown A. J.; Jessup W. Oxysterols: Sources, Cellular Storage and Metabolism, and New Insights into Their Roles in Cholesterol Homeostasis. Mol. Aspects Med. 2009, 30 (3), 111–122. 10.1016/j.mam.2009.02.005. 19248801

[ref34] Vejux A.; Malvitte L.; Lizard G. Side Effects of Oxysterols: Cytotoxicity, Oxidation, Inflammation, and Phospholipidosis. Braz. J. Med. Biol. Res. 2008, 41 (7), 545–556. 10.1590/S0100-879X2008000700001. 18719735

[ref35] Paradis S.; Leoni V.; Caccia C.; Berdeaux A.; Morin D. Cardioprotection by the TSPO Ligand 4′-Chlorodiazepam Is Associated with Inhibition of Mitochondrial Accumulation of Cholesterol at Reperfusion. Cardiovasc. Res. 2013, 98 (3), 420–427. 10.1093/cvr/cvt079. 23554458

[ref36] Musman J.; Paradis S.; Panel M.; Pons S.; Barau C.; Caccia C.; Leoni V.; Ghaleh B.; Morin D. A TSPO Ligand Prevents Mitochondrial Sterol Accumulation and Dysfunction during Myocardial Ischemia-Reperfusion in Hypercholesterolemic Rats. Biochem. Pharmacol. 2017, 142, 87–95. 10.1016/j.bcp.2017.06.125. 28645478

[ref37] McCommis K. S.; McGee A. M.; Laughlin M. H.; Bowles D. K.; Baines C. P. Hypercholesterolemia Increases Mitochondrial Oxidative Stress and Enhances the MPT Response in the Porcine Myocardium: Beneficial Effects of Chronic Exercise. Am. J. Physiol. Regul. Integr. Comp. Physiol. 2011, 301 (5), R1250–R1258. 10.1152/ajpregu.00841.2010. 21865543 PMC3213933

[ref38] Andreadou I.; Iliodromitis E. K.; Lazou A.; Görbe A.; Giricz Z.; Schulz R.; Ferdinandy P. Effect of Hypercholesterolaemia on Myocardial Function, Ischaemia-Reperfusion Injury and Cardioprotection by Preconditioning, Postconditioning and Remote Conditioning. Br. J. Pharmacol. 2017, 174 (12), 1555–1569. 10.1111/bph.13704. 28060997 PMC5446572

[ref39] Rosenberg P. VDAC2 as a Novel Target for Heart Failure: Ca2+ at the Sarcomere, Mitochondria and SR. Cell Calcium 2022, 104, 102586 10.1016/j.ceca.2022.102586. 35429733 PMC9459362

[ref40] Kwong J. Q.; Molkentin J. D. Physiological and Pathological Roles of the Mitochondrial Permeability Transition Pore in the Heart. Cell Metab 2015, 21 (2), 206–214. 10.1016/j.cmet.2014.12.001. 25651175 PMC4616258

[ref41] Briston T.; Selwood D. L.; Szabadkai G.; Duchen M. R. Mitochondrial Permeability Transition: A Molecular Lesion with Multiple Drug Targets. Trends Pharmacol. Sci. 2019, 40 (1), 50–70. 10.1016/j.tips.2018.11.004. 30527591

[ref42] Fauconnier J.; Roberge S.; Saint N.; Lacampagne A. Type 2 Ryanodine Receptor: A Novel Therapeutic Target in Myocardial Ischemia/Reperfusion. Pharmacol Ther 2013, 138 (3), 323–332. 10.1016/j.pharmthera.2013.01.015. 23384595

[ref43] Cohen M. V.; Yang X. M.; Downey J. M. The PH Hypothesis of Postconditioning: Staccato Reperfusion Reintroduces Oxygen and Perpetuates Myocardial Acidosis. Circulation 2007, 115 (14), 1895–1903. 10.1161/CIRCULATIONAHA.106.675710. 17389262

[ref44] Halestrap A. P.; Clarke S. J.; Javadov S. A. Mitochondrial Permeability Transition Pore Opening during Myocardial Reperfusion--a Target for Cardioprotection. Cardiovasc. Res. 2004, 61 (3), 372–385. 10.1016/S0008-6363(03)00533-9. 14962470

[ref45] Halestrap A. P. What Is the Mitochondrial Permeability Transition Pore?. J. Mol. Cell Cardiol 2009, 46 (6), 821–831. 10.1016/j.yjmcc.2009.02.021. 19265700

[ref46] Crompton M.; Costi A.; Hayat L. Evidence for the Presence of a Reversible Ca2+-Dependent Pore Activated by Oxidative Stress in Heart Mitochondria. Biochem. J. 1987, 245 (3), 915–918. 10.1042/bj2450915. 3117053 PMC1148218

[ref47] CROMPTON M.; COSTI A. Kinetic Evidence for a Heart Mitochondrial Pore Activated by Ca2+, Inorganic Phosphate and Oxidative Stress. A Potential Mechanism for Mitochondrial Dysfunction during Cellular Ca2+ Overload. Eur. J. Biochem. 1988, 178 (2), 489–501. 10.1111/j.1432-1033.1988.tb14475.x. 2850179

[ref48] Clarke S. J.; McStay G. P.; Halestrap A. P. Sanglifehrin A Acts as a Potent Inhibitor of the Mitochondrial Permeability Transition and Reperfusion Injury of the Heart by Binding to Cyclophilin-D at a Different Site from Cyclosporin A. J. Biol. Chem. 2002, 277 (38), 34793–34799. 10.1074/jbc.M202191200. 12095984

[ref49] Griffiths E. J.; Halestrap A. P. Mitochondrial Non-Specific Pores Remain Closed during Cardiac Ischaemia, but Open upon Reperfusion. Biochem. J. 1995, 307 (1), 93–98. 10.1042/bj3070093. 7717999 PMC1136749

[ref50] Griffiths E. J.; Halestrap A. P. Protection by Cyclosporin A of Ischemia/Reperfusion-Induced Damage in Isolated Rat Hearts. J. Mol. Cell Cardiol 1993, 25 (12), 1461–1469. 10.1006/jmcc.1993.1162. 7512654

[ref51] Hausenloy D. J.; Duchen M. R.; Yellon D. M. Inhibiting Mitochondrial Permeability Transition Pore Opening at Reperfusion Protects against Ischaemia-Reperfusion Injury. Cardiovasc. Res. 2003, 60 (3), 617–625. 10.1016/j.cardiores.2003.09.025. 14659807

[ref52] Argaud L.; Gateau-Roesch O.; Muntean D.; Chalabreysse L.; Loufouat J.; Robert D.; Ovize M. Specific Inhibition of the Mitochondrial Permeability Transition Prevents Lethal Reperfusion Injury. J. Mol. Cell Cardiol 2005, 38 (2), 367–374. 10.1016/j.yjmcc.2004.12.001. 15698843

[ref53] Connern C. P.; Halestrap A. P. Purification and N-Terminal Sequencing of Peptidyl-Prolyl Cis-Trans-Isomerase from Rat Liver Mitochondrial Matrix Reveals the Existence of a Distinct Mitochondrial Cyclophilin. Biochem. J. 1992, 284 (2), 381–385. 10.1042/bj2840381. 1599421 PMC1132649

[ref54] Beutner G.; Alanzalon R. E.; Porter G. A. Cyclophilin D Regulates the Dynamic Assembly of Mitochondrial ATP Synthase into Synthasomes. Sci. Rep 2017, 7 (1), 14488 10.1038/s41598-017-14795-x. 29101324 PMC5670235

[ref55] Leducq N.; Bono F.; Sulpice T.; Vin V.; Janiak P.; Le Fur G.; O’Connor S. E.; Herbert J. M. Role of Peripheral Benzodiazepine Receptors in Mitochondrial, Cellular, and Cardiac Damage Induced by Oxidative Stress and Ischemia-Reperfusion. J. Pharmacol Exp Ther 2003, 306 (3), 828–837. 10.1124/jpet.103.052068. 12928523

[ref56] Obame F. N.; Zini R.; Souktani R.; Berdeaux A.; Morin D. Peripheral Benzodiazepine Receptor-Induced Myocardial Protection Is Mediated by Inhibition of Mitochondrial Membrane Permeabilization. J. Pharmacol Exp Ther 2007, 323 (1), 336–345. 10.1124/jpet.107.124255. 17640950

[ref57] Kunduzova O. R.; Escourrou G.; De La Farge F.; Salvayre R.; Séguélas M. H.; Leducq N.; Bono F.; Herbert J. M.; Parini A. Involvement of Peripheral Benzodiazepine Receptor in the Oxidative Stress, Death-Signaling Pathways, and Renal Injury Induced by Ischemia-Reperfusion. J. Am. Soc. Nephrol 2004, 15 (8), 2152–2160. 10.1097/01.ASN.0000133563.41148.74. 15284300

[ref58] Gonzalez-Polo R. A.; Carvalho G.; Braun T.; Decaudin D.; Fabre C.; Larochette N.; Perfettini J. L.; Djavaheri-Mergny M.; Youlyouz-Marfak I.; Codogno P.; Raphael M.; Feuillard J.; Kroemer G. PK11195 Potently Sensitizes to Apoptosis Induction Independently from the Peripheral Benzodiazepin Receptor. Oncogene 2005, 24 (51), 7503–7513. 10.1038/sj.onc.1208907. 16091749

[ref59] Hans G.; Wislet-Gendebien S.; Lallemend F.; Robe P.; Rogister B.; Belachew S.; Nguyen L.; Malgrange B.; Moonen G.; Rigo J. M. Peripheral Benzodiazepine Receptor (PBR) Ligand Cytotoxicity Unrelated to PBR Expression. Biochem. Pharmacol. 2005, 69 (5), 819–830. 10.1016/j.bcp.2004.11.029. 15710359

[ref60] Fennell D. A.; Corbo M.; Pallaska A.; Cotter F. E. Bcl-2 Resistant Mitochondrial Toxicity Mediated by the Isoquinoline Carboxamide PK11195 Involves de Novo Generation of Reactive Oxygen Species. Br. J. Cancer 2001, 84 (10), 1397–1404. 10.1054/bjoc.2001.1788. 11355954 PMC2363650

[ref61] Scarf A. M.; Ittner L. M.; Kassiou M. The Translocator Protein (18 KDa): Central Nervous System Disease and Drug Design. J. Med. Chem. 2009, 52 (3), 581–592. 10.1021/jm8011678. 19133775

[ref62] Shoshan-Barmatz V.; De Pinto V.; Zweckstetter M.; Raviv Z.; Keinan N.; Arbel N. VDAC, a Multi-Functional Mitochondrial Protein Regulating Cell Life and Death. Mol. Aspects Med. 2010, 31 (3), 227–285. 10.1016/j.mam.2010.03.002. 20346371

[ref63] Veenman L.; Shandalov Y.; Gavish M. VDAC Activation by the 18 KDa Translocator Protein (TSPO), Implications for Apoptosis. J. Bioenerg Biomembr 2008, 40 (3), 199–205. 10.1007/s10863-008-9142-1. 18670869

[ref64] Gatliff J.; East D.; Crosby J.; Abeti R.; Harvey R.; Craigen W.; Parker P.; Campanella M. TSPO Interacts with VDAC1 and Triggers a ROS-Mediated Inhibition of Mitochondrial Quality Control. Autophagy 2014, 10 (12), 2279–2296. 10.4161/15548627.2014.991665. 25470454 PMC4502750

[ref65] Brdiczka D. G.; Zorov D. B.; Sheu S. S. Mitochondrial Contact Sites: Their Role in Energy Metabolism and Apoptosis. Biochim. Biophys. Acta 2006, 1762 (2), 148–163. 10.1016/j.bbadis.2005.09.007. 16324828

[ref66] Beavis A. D.; Garlid K. D. Inhibition of the Mitochondrial Inner Membrane Anion Channel by Dicyclohexylcarbodiimide. Evidence for a Specific Transport Pathway. J. Biol. Chem. 1988, 263 (16), 7574–7580. 10.1016/S0021-9258(18)68538-2. 2453508

[ref67] O’Rourke B. Pathophysiological and Protective Roles of Mitochondrial Ion Channels. J. Physiol 2000, 529 (1), 23–36. 10.1111/j.1469-7793.2000.00023.x. 11080248 PMC2270186

[ref68] Akar F. G.; Aon M. A.; Tomaselli G. F.; O’Rourke B. The Mitochondrial Origin of Postischemic Arrhythmias. J. Clin Invest 2005, 115 (12), 3527–3535. 10.1172/JCI25371. 16284648 PMC1280968

[ref69] Zorov D. B.; Filburn C. R.; Klotz L. O.; Zweier J. L.; Sollott S. J. Reactive Oxygen Species (ROS)-Induced ROS Release: A New Phenomenon Accompanying Induction of the Mitochondrial Permeability Transition in Cardiac Myocytes. J. Exp Med. 2000, 192 (7), 1001–1014. 10.1084/jem.192.7.1001. 11015441 PMC2193314

[ref70] Vanden Hoek T. L.; Becker L. B.; Shao Z.; Li C.; Schumacker P. T. Reactive Oxygen Species Released from Mitochondria during Brief Hypoxia Induce Preconditioning in Cardiomyocytes. J. Biol. Chem. 1998, 273 (29), 18092–18098. 10.1074/jbc.273.29.18092. 9660766

[ref71] Cortassa S.; Aon M. A.; Winslow R. L.; O’Rourke B. A Mitochondrial Oscillator Dependent on Reactive Oxygen Species. Biophys. J. 2004, 87 (3), 2060–2073. 10.1529/biophysj.104.041749. 15345581 PMC1304608

[ref72] Aon M. A.; Cortassa S.; Marbán E.; O’Rourke B. Synchronized Whole Cell Oscillations in Mitochondrial Metabolism Triggered by a Local Release of Reactive Oxygen Species in Cardiac Myocytes. J. Biol. Chem. 2003, 278 (45), 44735–44744. 10.1074/jbc.M302673200. 12930841

[ref73] Motloch L. J.; Hu J.; Akar F. G. The Mitochondrial Translocator Protein and Arrhythmogenesis in Ischemic Heart Disease. Oxid Med. Cell Longev 2015, 2015, 234104 10.1155/2015/234104. 25918579 PMC4397036

[ref74] Aon M. A.; Cortassa S.; Maack C.; O’Rourke B. Sequential Opening of Mitochondrial Ion Channels as a Function of Glutathione Redox Thiol Status. J. Biol. Chem. 2007, 282 (30), 21889–21900. 10.1074/jbc.M702841200. 17540766 PMC2292488

[ref75] Biary N.; Xie C.; Kauffman J.; Akar F. G. Biophysical Properties and Functional Consequences of Reactive Oxygen Species (ROS)-Induced ROS Release in Intact Myocardium. J. Physiol 2011, 589 (21), 5167–5179. 10.1113/jphysiol.2011.214239. 21825030 PMC3225672

[ref76] Brown D. A.; Aon M. A.; Akar F. G.; Liu T.; Sorarrain N.; O’Rourke B. Effects of 4′-Chlorodiazepam on Cellular Excitation-Contraction Coupling and Ischaemia-Reperfusion Injury in Rabbit Heart. Cardiovasc. Res. 2008, 79 (1), 141–149. 10.1093/cvr/cvn053. 18304929 PMC2562874

[ref77] Beavis A. D. Properties of the Inner Membrane Anion Channel in Intact Mitochondria. J. Bioenerg Biomembr 1992, 24 (1), 77–90. 10.1007/BF00769534. 1380509

[ref78] Ponikowski P.; Voors A. A.; Anker S. D.; Bueno H.; Cleland J. G. F.; Coats A. J. S.; Falk V.; Gonzalez-Juanatey J. R.; Harjola V.-P.; Jankowska E. A.; Jessup M.; Linde C.; Nihoyannopoulos P.; Parissis J. T.; Pieske B.; Riley J. P.; Rosano G. M. C.; Ruilope L. M.; Ruschitzka F.; Rutten F. H.; van der Meer P.; 2016 ESC Guidelines for the Diagnosis and Treatment of Acute and Chronic Heart Failure: The Task Force for the Diagnosis and Treatment of Acute and Chronic Heart Failure of the European Society of Cardiology (ESC)Developed with the Special Contribution of the Heart Failure Association (HFA) of the ESC. Eur. Heart J. 2016, 37 (27), 2129–2200. 10.1093/eurheartj/ehw128. 27206819

[ref79] Bax J. J.; Delgado V. Myocardial Viability as Integral Part of the Diagnostic and Therapeutic Approach to Ischemic Heart Failure. J. Nucl. Cardiol 2015, 22 (2), 229–245. 10.1007/s12350-015-0096-5. 25733105 PMC4490177

[ref80] Farber G.; Boczar K. E.; Wiefels C. C.; Zelt J. G. E.; Guler E. C.; deKemp R. A.; Beanlands R. S.; Rotstein B. H. The Future of Cardiac Molecular Imaging. Semin Nucl. Med. 2020, 50 (4), 367–385. 10.1053/j.semnuclmed.2020.02.005. 32540033

[ref81] Maddahi J.; Agostini D.; Bateman T. M.; Bax J. J.; Beanlands R. S. B.; Berman D. S.; Dorbala S.; Garcia E. V.; Feldman J.; Heller G. V.; Knuuti J. M.; Martinez-Clark P.; Pelletier-Galarneau M.; Shepple B.; Tamaki N.; Tranquart F.; Udelson J. E. Flurpiridaz F-18 PET Myocardial Perfusion Imaging in Patients With Suspected Coronary Artery Disease. J. Am. Coll Cardiol 2023, 82 (16), 1598–1610. 10.1016/j.jacc.2023.08.016. 37821170

[ref82] Viviano M.; Barresi E.; Siméon F. G.; Costa B.; Taliani S.; Da Settimo F.; Pike V. W.; Castellano S. Essential Principles and Recent Progress in the Development of TSPO PET Ligands for Neuroinflammation Imaging. Curr. Med. Chem. 2022, 29 (28), 4862–4890. 10.2174/0929867329666220329204054. 35352645 PMC10080361

[ref83] Hellberg S.; Liljenbäck H.; Eskola O.; Morisson-Iveson V.; Morrison M.; Trigg W.; Saukko P.; Ylä-Herttuala S.; Knuuti J.; Saraste A.; Roivainen A. Positron Emission Tomography Imaging of Macrophages in Atherosclerosis with 18 F-GE-180, a Radiotracer for Translocator Protein (TSPO). Contrast Media Mol. Imaging 2018, 2018, 9186902 10.1155/2018/9186902. 29950954 PMC5987326

[ref84] Luu T. G.; Kim H. K. 18F-Radiolabeled Translocator Protein (TSPO) PET Tracers: Recent Development of TSPO Radioligands and Their Application to PET Study. Pharmaceutics 2022, 14 (11), 2545 10.3390/pharmaceutics14112545. 36432736 PMC9697781

[ref85] Camsonne R.; Crouzel C.; Comar D.; Mazière M.; Prenant C.; Sastre J.; Moulin M.; Syrota A. Synthesis of N-(11C) Methyl, N-(Methyl-1 Propyl), (Chloro-2 Phenyl)-1 Isoquinoleine Carboxamide-3 (PK 11195): A New Ligand for Peripheral Benzodiazepine Receptors. J. Labelled Comp Radiopharm 1984, 21 (10), 985–991. 10.1002/jlcr.2580211012.

[ref86] Gaemperli O.; Shalhoub J.; Owen D. R. J.; Lamare F.; Johansson S.; Fouladi N.; Davies A. H.; Rimoldi O. E.; Camici P. G. Imaging Intraplaque Inflammation in Carotid Atherosclerosis with 11C-PK11195 Positron Emission Tomography/Computed Tomography. Eur. Heart J. 2012, 33 (15), 1902–1910. 10.1093/eurheartj/ehr367. 21933781

[ref87] Kobiyama K.; Ley K. Atherosclerosis. Circ. Res. 2018, 123 (10), 1118–1120. 10.1161/CIRCRESAHA.118.313816. 30359201 PMC6298754

[ref88] Hansson G. K.; Libby P.; Tabas I. Inflammation and Plaque Vulnerability. J. Intern Med. 2015, 278 (5), 483–493. 10.1111/joim.12406. 26260307 PMC5082111

[ref89] Zavala F.; Haumont J.; Lenfant M. Interaction of Benzodiazepines with Mouse Macrophages. Eur. J. Pharmacol. 1984, 106 (3), 561–566. 10.1016/0014-2999(84)90059-1. 6151510

[ref90] Laitinen I.; Marjamäki P.; Någren K.; Laine V. J. O.; Wilson I.; Leppänen P.; Ylä-Herttuala S.; Roivainen A.; Knuuti J. Uptake of Inflammatory Cell Marker [11C]PK11195 into Mouse Atherosclerotic Plaques. Eur. J. Nucl. Med. Mol. Imaging 2009, 36 (1), 73–80. 10.1007/s00259-008-0919-6. 18712383

[ref91] Tegler G.; Sörensen J.; Ericson K.; Björck M.; Wanhainen A. 4D-PET/CT with [ 11 C]-PK11195 and [ 11 C]-D-Deprenyl Does Not Identify the Chronic Inflammation in Asymptomatic Abdominal Aortic Aneurysms. European Journal of Vascular & Endovascular Surgery 2013, 45, 351–356. 10.1016/j.ejvs.2013.01.011. 23394769

[ref92] Pugliese F.; Gaemperli O.; Kinderlerer A. R.; Lamare F.; Shalhoub J.; Davies A. H.; Rimoldi O. E.; Mason J. C.; Camici P. G. Imaging of Vascular Inflammation with [11C]-PK11195 and Positron Emission Tomography/Computed Tomography Angiography. J. Am. Coll Cardiol 2010, 56 (8), 653–661. 10.1016/j.jacc.2010.02.063. 20705222

[ref93] Gulati A.; Bagga A. Large Vessel Vasculitis. Pediatr Nephrol 2010, 25 (6), 1037 10.1007/s00467-009-1312-9. 19844748 PMC2855435

[ref94] Shah F.; Hume S. P.; Pike V. W.; Ashworth S.; McDermott J. Synthesis of the Enantiomers of [N-Methyl-11C]PK 11195 and Comparison of Their Behaviours as Radioligands for PK Binding Sites in Rats. Nucl. Med. Biol. 1994, 21 (4), 573–581. 10.1016/0969-8051(94)90022-1. 9234314

[ref95] Dixon T.; Uyar A.; Ferguson-Miller S.; Dickson A. Membrane-Mediated Ligand Unbinding of the PK-11195 Ligand from TSPO. Biophys. J. 2021, 120 (1), 158–167. 10.1016/j.bpj.2020.11.015. 33221248 PMC7820730

[ref96] Zhang L.; Hu K.; Shao T.; Hou L.; Zhang S.; Ye W.; Josephson L.; Meyer J. H.; Zhang M. R.; Vasdev N.; Wang J.; Xu H.; Wang L.; Liang S. H. Recent Developments on PET Radiotracers for TSPO and Their Applications in Neuroimaging. Acta Pharm. Sin B 2021, 11 (2), 373–393. 10.1016/j.apsb.2020.08.006. 33643818 PMC7893127

[ref97] Cuhlmann S.; Gsell W.; Van Der Heiden K.; Habib J.; Tremoleda J. L.; Khalil M.; Turkheimer F.; Meens M. J.; Kwak B. R.; Bird J.; Davenport A. P.; Clark J.; Haskard D.; Krams R.; Jones H.; Evans P. C. In Vivo Mapping of Vascular Inflammation Using the Translocator Protein Tracer 18 F-FEDAA1106. Mol. Imaging 2014, 13, 7290.2014.00014 10.2310/7290.2014.00014. 24825602

[ref98] Hellberg S.; Silvola J. M. U.; Kiugel M.; Liljenbäck H.; Savisto N.; Li X. G.; Thiele A.; Lehmann L.; Heinrich T.; Vollmer S.; Hakovirta H.; Laine V. J. O.; Ylä-Herttuala S.; Knuuti J.; Roivainen A.; Saraste A. 18-KDa Translocator Protein Ligand 18F-FEMPA: Biodistribution and Uptake into Atherosclerotic Plaques in Mice. J. Nucl. Cardiol 2017, 24 (3), 862–871. 10.1007/s12350-016-0527-y. 27225517

[ref99] Perrone M.; Moon B. S.; Park H. S.; Laquintana V.; Jung J. H.; Cutrignelli A.; Lopedota A.; Franco M.; Kim S. E.; Lee B. C.; Denora N. A Novel PET Imaging Probe for the Detection and Monitoring of Translocator Protein 18 KDa Expression in Pathological Disorders. Sci. Rep 2016, 6, 20422 10.1038/srep20422. 26853260 PMC4745082

[ref100] Kim G. R.; Paeng J. C.; Jung J. H.; Moon B. S.; Lopalco A.; Denora N.; Lee B. C.; Kim S. E. Assessment of TSPO in a Rat Experimental Autoimmune Myocarditis Model: A Comparison Study between [18F]Fluoromethyl-PBR28 and [18F]CB251. Int. J. Mol. Sci. 2018, 19 (1), 276 10.3390/ijms19010276. 29342117 PMC5796222

[ref101] Sagar S.; Liu P. P.; Cooper L. T. Myocarditis. Lancet 2012, 379 (9817), 738–747. 10.1016/S0140-6736(11)60648-X. 22185868 PMC5814111

[ref102] Laissy J. P.; Hyafil F.; Feldman L. J.; Juliard J. M.; Schouman-Claeys E.; Steg P. G.; Faraggi M. Differentiating Acute Myocardial Infarction from Myocarditis: Diagnostic Value of Early- and Delayed-Perfusion Cardiac MR Imaging. Radiology 2005, 237 (1), 75–82. 10.1148/radiol.2371041322. 16126925

[ref103] Angelini A.; Calzolari V.; Calabrese F.; Boffa G. M.; Maddalena F.; Chioin R.; Thiene G. Myocarditis Mimicking Acute Myocardial Infarction: Role of Endomyocardial Biopsy in the Differential Diagnosis. Heart 2000, 84 (3), 245–250. 10.1136/heart.84.3.245. 10956283 PMC1760950

[ref104] Maekawa K.; Tsuji A. B.; Yamashita A.; Sugyo A.; Katoh C.; Tang M.; Nishihira K.; Shibata Y.; Koshimoto C.; Zhang M. R.; Nishii R.; Yoshinaga K.; Asada Y. Translocator Protein Imaging with 18F-FEDAC-Positron Emission Tomography in Rabbit Atherosclerosis and Its Presence in Human Coronary Vulnerable Plaques. Atherosclerosis 2021, 337, 7–17. 10.1016/j.atherosclerosis.2021.10.003. 34662838

[ref105] Luo R.; Wang L.; Ye F.; Wang Y.-R.; Fang W.; Zhang M.-R.; Wang F. [18F]FEDAC Translocator Protein Positron Emission Tomography-Computed Tomography for Early Detection of Mitochondrial Dysfunction Secondary to Myocardial Ischemia. Ann. Nucl. Med. 2021, 35, 927–936. 10.1007/s12149-021-01630-7. 34081287 PMC8285353

[ref106] Mou T.; Tian J.; Tian Y.; Yun M.; Li J.; Dong W.; Lu X.; Zhu Z.; Mi H.; Zhang X.; Li X. Automated Synthesis and Preliminary Evaluation of [ 18 F] FDPA for Cardiac Inflammation Imaging in Rats after Myocardial Infarction. Sci. Rep. 2020, 10, 18685 10.1038/s41598-020-75705-2. 33122775 PMC7596090

[ref107] Turkheimer F. E.; Rizzo G.; Bloomfield P. S.; Howes O.; Zanotti-Fregonara P.; Bertoldo A.; Veronese M. The Methodology of TSPO Imaging with Positron Emission Tomography. Biochem. Soc. Trans. 2015, 43 (4), 586–592. 10.1042/BST20150058. 26551697 PMC4613512

[ref108] Owen D. R.; Howell O. W.; Tang S. P.; Wells L. A.; Bennacef I.; Bergstrom M.; Gunn R. N.; Rabiner E. A.; Wilkins M. R.; Reynolds R.; Matthews P. M.; Parker C. A. Two Binding Sites for [3H]PBR28 in Human Brain: Implications for TSPO PET Imaging of Neuroinflammation. J. Cereb Blood Flow Metab 2010, 30 (9), 1608–1618. 10.1038/jcbfm.2010.63. 20424634 PMC2949260

[ref109] Kreisl W. C.; Fujita M.; Fujimura Y.; Kimura N.; Jenko K. J.; Kannan P.; Hong J.; Morse C. L.; Zoghbi S. S.; Gladding R. L.; Jacobson S.; Oh U.; Pike V. W.; Innis R. B. Comparison of [(11)C]-(R)-PK 11195 and [(11)C]PBR28, Two Radioligands for Translocator Protein (18 KDa) in Human and Monkey: Implications for Positron Emission Tomographic Imaging of This Inflammation Biomarker. Neuroimage 2010, 49 (4), 2924–2932. 10.1016/j.neuroimage.2009.11.056. 19948230 PMC2832854

[ref110] Wang M.; Gao M.; Hutchins G. D.; Zheng Q. H. Synthesis of [11C]FEDAA1106 as a New PET Imaging Probe of Peripheral Benzodiazepine Receptor Expression. Eur. J. Med. Chem. 2009, 44 (6), 2748–2753. 10.1016/j.ejmech.2008.08.001. 18790550

[ref111] Moon B. S.; Kim B. S.; Park C.; Jung J. H.; Lee Y. W.; Lee H. Y.; Chi D. Y.; Lee B. C.; Kim S. E. [(18)F]Fluoromethyl-PBR28 as a Potential Radiotracer for TSPO: Preclinical Comparison with [(11)C]PBR28 in a Rat Model of Neuroinflammation. Bioconjug Chem. 2014, 25 (2), 442–450. 10.1021/bc400556h. 24400917

[ref112] Kreisl W. C.; Lyoo C. H.; Liow J.-S.; Wei M.; Snow J.; Page E.; Jenko K. J.; Morse C. L.; Zoghbi S. S.; Pike V. W.; Turner R. S.; Innis R. B. 1 C-PBR28 Binding to Translocator Protein Increases with Progression of Alzheimer’s Disease HHS Public Access. Neurobiol. Aging 2016, 44, 53–61. 10.1016/j.neurobiolaging.2016.04.011. 27318133 PMC4939713

[ref113] Briard E.; Zoghbi S. S.; Imaizumi M.; Gourley J. P.; Shetty H. U.; Hong J.; Cropley V.; Fujita M.; Innis R. B.; Pike V. W. Synthesis and Evaluation in Monkey of Two Sensitive 11C-Labeled Aryloxyanilide Ligands for Imaging Brain Peripheral Benzodiazepine Receptors in Vivo. J. Med. Chem. 2008, 51 (1), 17–30. 10.1021/jm0707370. 18067245

[ref114] Imaizumi M.; Briard E.; Zoghbi S. S.; Gourley J. P.; Hong J.; Musachio J. L.; Gladding R.; Pike V. W.; Innis R. B.; Fujita M. Kinetic Evaluation in Nonhuman Primates of Two New PET Ligands for Peripheral Benzodiazepine Receptors in Brain. Synapse 2007, 61 (8), 595–605. 10.1002/syn.20394. 17455247

[ref115] Damont A.; Médran-Navarrete V.; Cacheux F.; Kuhnast B.; Pottier G.; Bernards N.; Marguet F.; Puech F.; Boisgard R.; Dollé F. Novel Pyrazolo[1,5-a]Pyrimidines as Translocator Protein 18 KDa (TSPO) Ligands: Synthesis, in Vitro Biological Evaluation, [18F]-Labeling, and in Vivo Neuroinflammation PET Images. J. Med. Chem. 2015, 58 (18), 7449–7464. 10.1021/acs.jmedchem.5b00932. 26280386

[ref116] Yanamoto K.; Kumata K.; Yamasaki T.; Odawara C.; Kawamura K.; Yui J.; Hatori A.; Suzuki K.; Zhang M. R. [18F]FEAC and [18F]FEDAC: Two Novel Positron Emission Tomography Ligands for Peripheral-Type Benzodiazepine Receptor in the Brain. Bioorg. Med. Chem. Lett. 2009, 19 (6), 1707–1710. 10.1016/j.bmcl.2009.01.093. 19217778

[ref117] Wickstrøm T.; Clarke A.; Gausemel I.; Horn E.; Jørgensen K.; Khan I.; Mantzilas D.; Rajanayagam T.; In ’T Veld D. J.; Trigg W. The Development of an Automated and GMP Compliant FASTlab Synthesis of [(18) F]GE-180; a Radiotracer for Imaging Translocator Protein (TSPO). J. Labelled Comp Radiopharm 2014, 57 (1), 42–48. 10.1002/jlcr.3112. 24448744

[ref118] MacAskill M. G.; Stadulyte A.; Williams L.; Morgan T. E. F.; Sloan N. L.; Alcaide-Corral C. J.; Walton T.; Wimberley C.; McKenzie C. A.; Spath N.; Mungall W.; BouHaidar R.; Dweck M. R.; Gray G. A.; Newby D. E.; Lucatelli C.; Sutherland A.; Pimlott S. L.; Tavares A. A. S. Quantification of Macrophage-Driven Inflammation During Myocardial Infarction with 18F-LW223, a Novel TSPO Radiotracer with Binding Independent of the Rs6971 Human Polymorphism. J. Nucl. Med. 2021, 62 (4), 536–544. 10.2967/jnumed.120.243600. 32859708 PMC8049364

[ref119] Holck M.; Osterrieder W. The Peripheral, High Affinity Benzodiazepine Binding Site Is Not Coupled to the Cardiac Ca2+ Channel. Eur. J. Pharmacol. 1985, 118 (3), 293–301. 10.1016/0014-2999(85)90140-2. 2417868

[ref120] Edoute Y.; Giris J.; Ben-Haim S. A.; Lochner A.; Weizman A.; Hayam G.; Katz Y.; Gavish M. Ro 5–4864 and PK 11195, but Not Diazepam, Depress Cardiac Function in an Isolated Working Rat Heart Model. Pharmacology 1993, 46 (4), 224–230. 10.1159/000139049. 8387216

[ref121] Mestre M.; Carriot T.; Belin C.; Uzan A.; Renault C.; Dubroeucq M. C.; Guérémy C.; Doble A.; Le Fur G. Electrophysiological and Pharmacological Evidence That Peripheral Type Benzodiazepine Receptors Are Coupled to Calcium Channels in the Heart. Life Sci. 1985, 36 (4), 391–400. 10.1016/0024-3205(85)90126-2. 2578209

[ref122] Saegusa K.; Furukawa Y.; Chiba S. Pharmacological Analyses of Hydralazne-Induced Cardiac Action in Intact Dogs and Isolated, Blood-Perfused Canine Atria. J. Cardiovasc Pharmacol 1986, 8 (3), 614–620. 10.1097/00005344-198605000-00026. 2425181

[ref123] Grupp I. L.; French J. F.; Matlib M. A. Benzodiazepine Ro 5–4864 Increases Coronary Flow. Eur. J. Pharmacol. 1987, 143 (1), 143–147. 10.1016/0014-2999(87)90746-1. 3691649

[ref124] Weissman B. A.; Bolger G. T.; Chiang P. K. Interactions between Nitrogen Oxide-Containing Compounds and Peripheral Benzodiazepine Receptors. FEBS Lett. 1990, 260 (2), 169–172. 10.1016/0014-5793(90)80095-Z. 1688811

[ref125] Leeuwin R. S.; Zeegers A.; Van Hamme J.; Van Wilgenburg H. Modification of Cardiac Actions of RO 05–4864 by PK 11195 and Flumazenil in the Perfused Rat Heart. Life Sci. 1997, 61 (17), 1631–1642. 10.1016/S0024-3205(97)00768-6. 9363978

[ref126] Xiao J.; Liang D.; Zhang H.; Liu Y.; Li F.; Chen Y. H. 4′-Chlorodiazepam, a Translocator Protein (18 KDa) Antagonist, Improves Cardiac Functional Recovery during Postischemia Reperfusion in Rats. Exp Biol. Med. (Maywood) 2010, 235 (4), 478–486. 10.1258/ebm.2009.009291. 20407080

[ref127] Terrovitis J.; Katsaros L.; Tsamatsoulis M.; Sventzouri S.; Sousonis V.; Bonios M.; Kapelios C.; Vakrou S.; Ntalianis A.; Papalois A.; O’Rourke B.; Nanas J. CARDIOPROTECTION BY STIMULATION OF MITOCHONDRIAL BENZODIAZEPINE RECEPTORS DURING REPERFUSION, IN A PORCINE ACUTE ISCHEMIA-REPERFUSION MODEL. J. Am. Coll. Cardiol. 2012, 59 (13), E545 10.1016/S0735-1097(12)60546-4.

[ref128] Tsamatsoulis M.; Kapelios C. J.; Katsaros L.; Vakrou S.; Sousonis V.; Sventzouri S.; Michelinakis N.; Perrea D. N.; Anastasiou-Nana M.; Malliaras K. Cardioprotective Effects of Intracoronary Administration of 4-Chlorodiazepam in Small and Large Animal Models of Ischemia-Reperfusion. Int. J. Cardiol 2016, 224, 90–95. 10.1016/j.ijcard.2016.09.011. 27643472

[ref129] Jaiswal A.; Kumar S.; Enjamoori R.; Seth S.; Dinda A. K.; Maulik S. K. Peripheral Benzodiazepine Receptor Ligand Ro5-4864 Inhibits Isoprenaline-Induced Cardiac Hypertrophy in Rats. Eur. J. Pharmacol. 2010, 644 (1–3), 146–153. 10.1016/j.ejphar.2010.06.058. 20621082

[ref130] Kenyon C. J.; Thomson I.; Fraser R. Stimulation of Aldosterone Secretion by Benzodiazepines in Bovine Adrenocortical Cells. Fundam Clin Pharmacol 1999, 13 (2), 213–219. 10.1111/j.1472-8206.1999.tb00341.x. 10226766

[ref131] Ilic A.; Todorovic D.; Mutavdzin S.; Boricic N.; Nedeljkovic B. B.; Stankovic S.; Simic T.; Stevanovic P.; Celic V.; Djuric D. Translocator Protein Modulation by 4′-Chlorodiazepam and NO Synthase Inhibition Affect Cardiac Oxidative Stress, Cardiometabolic and Inflammatory Markers in Isoprenaline-Induced Rat Myocardial Infarction. Int. J. Mol. Sci. 2021, 22 (6), 2867 10.3390/ijms22062867. 33799869 PMC8000569

[ref132] de Tassigny A. d. A.; Assaly R.; Schaller S.; Pruss R. M.; Berdeaux A.; Morin D. Mitochondrial Translocator Protein (TSPO) Ligands Prevent Doxorubicin-Induced Mechanical Dysfunction and Cell Death in Isolated Cardiomyocytes. Mitochondrion 2013, 13 (6), 688–697. 10.1016/j.mito.2013.10.001. 24121045

[ref133] Le Fur G.; Vaucher N.; Perrier M. L.; Flamier A.; Benavides J.; Renault C.; Dubroeucq M. C.; Guérémy C.; Uzan A. Differentiation between Two Ligands for Peripheral Benzodiazepine Binding Sites, [3H]RO5–4864 and [3H]PK 11195, by Thermodynamic Studies. Life Sci. 1983, 33 (5), 449–457. 10.1016/0024-3205(83)90794-4. 6308375

[ref134] Lheureux P.; Vranckx M.; Leduc D.; Askenasi R. Effect of PK11195 on Cardiovascular Toxicity Due to Verapamil: An Experimental Study in the Dog. J. Toxicol. Clin. Exp. 1990, 10 (7-8), 449–460. 2135060

[ref135] Li J.; Wang J.; Zeng Y. Peripheral Benzodiazepine Receptor Ligand, PK11195 Induces Mitochondria Cytochrome c Release and Dissipation of Mitochondria Potential via Induction of Mitochondria Permeability Transition. Eur. J. Pharmacol. 2007, 560 (2–3), 117–122. 10.1016/j.ejphar.2006.12.027. 17291492

[ref136] Schild L.; Keilhoff G.; Augustin W.; Reiser G.; Striggow F. Distinct Ca2+ Thresholds Determine Cytochrome c Release or Permeability Transition Pore Opening in Brain Mitochondria. FASEB J. 2001, 15 (3), 565–567. 10.1096/fj.00-0551fje. 11259368

[ref137] Campiani G.; Fiorini I.; De Filippis M. P.; Ciani S. M.; Garofalo A.; Nacci V.; Giorgi G.; Sega A.; Botta M.; Chiarini A.; Budriesi R.; Bruni G.; Romeo M. R.; Manzoni C.; Mennini T. Cardiovascular Characterization of Pyrrolo[2,1-d][1,5]Benzothiazepine Derivatives Binding Selectively to the Peripheral-Type Benzodiazepine Receptor (PBR): From Dual PBR Affinity and Calcium Antagonist Activity to Novel and Selective Calcium Entry Blockers. J. Med. Chem. 1996, 39 (15), 2922–2938. 10.1021/jm960162z. 8709127

[ref138] Mestre M.; Bouetard G.; Uzan A.; Gueremy C.; Renault C.; Dubroeucq M. C.; Le Fur G. PK 11195, an Antagonist of Peripheral Benzodiazepine Receptors, Reduces Ventricular Arrhythmias during Myocardial Ischemia and Reperfusion in the Dog. Eur. J. Pharmacol. 1985, 112 (2), 257–260. 10.1016/0014-2999(85)90505-9. 2992998

[ref139] Seidlmayer L. K.; Hanson B. J.; Thai P. N.; Schaefer S.; Bers D. M.; Dedkova E. N. PK11195 Protects From Cell Death Only When Applied During Reperfusion: Succinate-Mediated Mechanism of Action. Front Physiol 2021, 12, 628508 10.3389/fphys.2021.628508. 34149440 PMC8212865

[ref140] Anversa P.; Olivetti G.; Leri A.; Liu Y.; Kajstura J. Myocyte Cell Death and Ventricular Remodeling. Curr. Opin Nephrol Hypertens 1997, 6 (2), 169–176. 10.1097/00041552-199703000-00011. 9146980

[ref141] Leducq-Alet N.; Vin V.; Savi P.; Bono F. TNF-Alpha Induced PMN Apoptosis in Whole Human Blood: Protective Effect of SSR180575, a Potent and Selective Peripheral Benzodiazepine Ligand. Biochem. Biophys. Res. Commun. 2010, 399 (4), 475–479. 10.1016/j.bbrc.2010.07.011. 20621066

[ref142] Bordet T.; Buisson B.; Michaud M.; Drouot C.; Galéa P.; Delaage P.; Akentieva N. P.; Evers A. S.; Covey D. F.; Ostuni M. A.; Lacapère J. J.; Massaad C.; Schumacher M.; Steidl E. M.; Maux D.; Delaage M.; Henderson C. E.; Pruss R. M. Identification and Characterization of Cholest-4-En-3-One, Oxime (TRO19622), a Novel Drug Candidate for Amyotrophic Lateral Sclerosis. J. Pharmacol Exp Ther 2007, 322 (2), 709–720. 10.1124/jpet.107.123000. 17496168

[ref143] Schaller S.; Paradis S.; Ngoh G. A.; Assaly R.; Buisson B.; Drouot C.; Ostuni M. A.; Lacapere J. J.; Bassissi F.; Bordet T.; Berdeaux A.; Jones S. P.; Morin D.; Pruss R. M. TRO40303, a New Cardioprotective Compound, Inhibits Mitochondrial Permeability Transition. J. Pharmacol Exp Ther 2010, 333 (3), 696–706. 10.1124/jpet.110.167486. 20215409

[ref144] Kim G. T.; Chun Y. S.; Park J. W.; Kim M. S. Role of Apoptosis-Inducing Factor in Myocardial Cell Death by Ischemia-Reperfusion. Biochem. Biophys. Res. Commun. 2003, 309 (3), 619–624. 10.1016/j.bbrc.2003.08.045. 12963035

[ref145] Hansson M. J.; Llwyd O.; Morin D.; De Paulis D.; Arnoux T.; Gouarné C.; Koul S.; Engblom H.; Bordet T.; Tissier R.; Arheden H.; Erlinge D.; Halestrap A. P.; Berdeaux A.; Pruss R. M.; Schaller S. Differences in the Profile of Protection Afforded by TRO40303 and Mild Hypothermia in Models of Cardiac Ischemia/Reperfusion Injury. Eur. J. Pharmacol. 2015, 760, 7–19. 10.1016/j.ejphar.2015.04.009. 25895640

[ref146] Pastorino J. G.; Hoek J. B. Regulation of Hexokinase Binding to VDAC. J. Bioenerg Biomembr 2008, 40 (3), 171–182. 10.1007/s10863-008-9148-8. 18683036 PMC2662512

[ref147] Morohaku K.; Pelton S. H.; Daugherty D. J.; Butler W. R.; Deng W.; Selvaraj V. Translocator Protein/Peripheral Benzodiazepine Receptor Is Not Required for Steroid Hormone Biosynthesis. Endocrinology 2014, 155 (1), 89–97. 10.1210/en.2013-1556. 24174323 PMC3868810

[ref148] Le Lamer S.; Paradis S.; Rahmouni H.; Chaimbault C.; Michaud M.; Culcasi M.; Afxantidis J.; Latreille M.; Berna P.; Berdeaux A.; Pietri S.; Morin D.; Donazzolo Y.; Abitbol J. L.; Pruss R. M.; Schaller S. Translation of TRO40303 from Myocardial Infarction Models to Demonstration of Safety and Tolerance in a Randomized Phase I Trial. J. Transl Med. 2014, 12 (1), 38 10.1186/1479-5876-12-38. 24507657 PMC3923730

[ref149] Rationale and Design of the “MITOCARE” Study: A Phase II, Multicenter, Randomized, Double-Blind, Placebo-Controlled Study to Assess the Safety and Efficacy of TRO40303 for the Reduction of Reperfusion Injury in Patients Undergoing Percutaneous Coronary Intervention for Acute Myocardial Infarction. Cardiology 2013, 123 (4), 201–207. 10.1159/000342981. 23202613

[ref150] BØtker H. E. Mitochondrial Care in Acute Myocardial Infarction. Eur. Heart J. 2015, 36 (2), 77–79. 10.1093/eurheartj/ehu380. 25179763

[ref151] Charbonneau P; Syrota A; Crouzel C; Valois J M; Prenant C; Crouzel M Peripheral-Type Benzodiazepine Receptors in the Living Heart Characterized by Positron Emission Tomography. Circulation 1986, 73 (3), 476–483. 10.1161/01.CIR.73.3.476. 3004781

